# Associations Between Self-reported Inhibitory Control, Stress, and Alcohol (Mis)use During the First Wave of the COVID-19 Pandemic in the UK: a National Cross-sectional Study Utilising Data From Four Birth Cohorts

**DOI:** 10.1007/s11469-021-00599-8

**Published:** 2021-08-03

**Authors:** James M. Clay, Lorenzo D. Stafford, Matthew O. Parker

**Affiliations:** 1 Department of Psychology, University of Portsmouth, King Henry Building, King Henry I Street, Portsmouth, PO1 2DY UK; 2 Brain and Behaviour Laboratory, School of Pharmacy and Biomedical Sciences, University of Portsmouth, White Swan Road, Portsmouth, PO1 2DT UK

**Keywords:** COVID-19, Alcohol, Stress, Inhibitory control, Impulsivity, Risk-taking

## Abstract

We explored (1) self-reported changes in alcohol use during the pandemic in the UK and (2) the extent to which self-reported inhibitory control and/or stress were associated with any change in drinking behaviour. We used a UK-based cross-sectional online survey administered to four nationally representative birth cohorts (*N* = 13,453). A significant minority of 30- (29.08%) and 50-year-olds (26.67%) reported drinking more, and between 32.23 and 45.02% of respondents reported feeling more stressed depending on the cohort. Stress was associated with hazardous drinking among 30-year-olds (OR = 3.77, 95% CI 1.15 to 12.28). Impatience was associated with both increased alcohol use (1.14, 95% CI 1.06, 1.24) and hazardous drinking (1.20, 95% CI 1.05, 1.38) among 19-year-olds. Risk-taking was associated with hazardous drinking for 30-year-olds (OR = 1.18, 95% CI 1.05, 1.32). These data highlight concerns for those at risk of alcohol misuse and alcohol-related harm during COVID-19 lockdowns.

**Supplementary Information:**

The online version contains supplementary material available at 10.1007/s11469-021-00599-8.

Since being first identified in Wuhan, China, in December 2019, severe acute respiratory syndrome coronavirus 2 (SARS-CoV-2), the virus that causes COVID-19, has caused a significant threat to global health (Sohrabi et al., 2020). Governments around the world responded by imposing ‘lockdowns’ (orders to remain at home, and socially isolate) on their populations, and available evidence supports this action as a means of mitigating the rate of spread of the virus (Anderson et al., 2020). However, the indirect impact of lockdown on public health has raised concern, particularly relating to mental health and well-being (Bhattacharjee & Acharya, 2020; Gavin et al., 2020; Ornell et al., 2020; Pfefferbaum & North, 2020).

Concerns that the lockdowns may increase alcohol misuse have been raised, particularly concerning people at high-risk of developing, or re-establishing, hazardous alcohol use (Clay & Parker, 2020; Finlay & Gilmore, 2020; Rehm et al., 2020). An example of individuals who are at high risk of alcohol misuse are people that display poor inhibitory control (Dalley & Ersche, 2019; Lee et al., 2019). Inhibitory control is generally conceptualised as one of the core executive functions (Diamond, 2013). It is a complex and multifaceted construct made up of several subcomponents: *response inhibition* (i.e., action inhibition, action cancellation), *sensitivity to delay* (i.e., delay discounting, patience), *sensitivity to risk/reward* (risk-taking, sensation seeking), and *attention* (i.e., capacity to focus and avoid interference) (Strickland & Johnson, 2020). Indeed, several lines of evidence from pre-clinical translational work (Belin et al., 2008; Kreek et al., 2005), neuroimaging studies (Bosker et al., 2017; Voon et al., 2020), and heritability studies (Ersche et al., 2010; Khemiri et al., 2016) converge to suggest that poor inhibitory control is both a risk factor for the development, and consequence, of substance misuse and addiction.

The association between stress and alcohol use is also well established (Jose et al., 2000; Ruisoto & Contador, 2019; Sinha, 2012). Similar to inhibitory control, stress plays a critical role in both the onset and maintenance of alcohol misuse and addiction (Becker, 2017). On the one hand, the acute anxiolytic properties of alcohol motivate some individuals to drink (Kwako & Koob, 2017). On the other, perhaps counterintuitively, alcohol acts as a physiological ‘stressor’: acute exposure to alcohol stimulates the hypothalamic-pituitary-adrenocortical (HPA) axis through direct activation of GABA_A_ receptors in the paraventricular nucleus (Armario, 2010). Finally, exposure to either chronic stress or chronic alcohol misuse both lead to blunted stress responses, including dysregulation of the HPA axis — a known risk factor for hazardous drinking and addiction (Milivojevic & Sinha, 2018).

Recently, we have demonstrated a complex interplay between inhibitory control, stress, and alcohol use, where an experimentally induced acute psychosocial stressor increased craving for alcohol (Clay et al., 2018), and voluntary alcohol consumption (Clay & Parker, 2018) in healthy (non-addicted) individuals. We found that the strength of these stress-induced increases in alcohol craving and consumption were predicated on individual differences in risk-taking personality traits, stress reactivity, and stress recovery. Collectively, our findings suggest these innate (e.g., poor inhibitory control), and environmental (e.g., ‘state’ induced stress) factors may combine to make particular individuals more at risk of alcohol misuse.

Here, we analysed the first sweep of the Centre for Longitudinal Studies (CLS) COVID-19 survey (University of London Institute of Education Centre for Longitudinal Studies, 2020a) — which was answered by individuals from five nationally representative cohorts who have been providing data since childhood — to explore (1) self-reported changes alcohol use during the pandemic in the UK and (2) the extent to which self-reported inhibitory control and/or stress were associated with any change in drinking behaviour.

## Methods

### Data Source

We used data from the first wave of the CLS COVID-19 survey (University of London Institute of Education Centre for Longitudinal Studies, 2020a). The survey design, recruitment procedure, and fieldwork processes have been described in detail elsewhere (Brown et al., 2020). Briefly, the survey was administered between 2 and 31 May 2020, using Qualtrics (Provo, Utah), to 50,479 individuals from five nationally representative UK birth cohorts. These included (1) the Millennium Cohort Study (MCS), who are part of ‘Generation Z’, and were aged 19; (2) Next Steps, who are part of the ‘Millennial’ generation, who were aged 30; (3) the 1970 British Cohort Study (BCS70), who belong to ‘Generation X’ — aged 50; (4) the National Child Development Study (NCDS), who were aged 62 and were born in the latter part of the ‘Baby Boomer’ generation; and (5) the National Study of Health and Development (NSHD), who were born at the beginning of the ‘Baby Boomer’ era, and were aged 74. Due to the nature of the survey, only those who had their email address previously recorded were approached. Overall, 18,042 of those invited responded, achieving a response rate (RR) of 35.7%. This response rate is similar to comparable national web surveys conducted at this time, such as the Understanding Society COVID-19 survey (Institute for Social & Economic Research, 2020). Ethnicity data was linked from previous survey waves (Kelly, 2008; University of London Institute of Education Centre for Longitudinal Studies, 2016, 2020b, 2020c). All data used in this study are available from the UK Data Service Website (https://ukdataservice.ac.uk/) under the ‘Safeguarded’ data access policy.

### Study Sample

Due to data availability at the time of analysis, four of the five cohorts included in the COVID-19 survey were analysed. Namely, the MCS cohort members (*n* = 2645, RR = 26.59%), Next Steps (*n* = 1907, RR = 20.33%), the BCS70 (*n* = 4223, RR = 40.38%), and the NCDS (*n* = 5,178, RR = 57.90%). The study was restricted to UK-based respondents; thus, emigrants (*n* = 500) were excluded prior to analysis. This left 13,453 cases for analysis. A detailed overview of the study sample is presented in the Supplementary Fig. 1. Selected sample characteristics are shown in Table 1.

**Table 1:** Selected demographic characteristics

Variable	MCS (*n* = 2644)			Next Steps (*n* = 1852)			BCS70 (*n* = 3997)			NCDS (*n* = 4960)		
	Statistic	LL	UL	Statistic	LL	UL	Statistic	LL	UL	Statistic	LL	UL
Age in years	19			30			50			62		
Sex, %												
Male	49.46	46.47	52.45	43.14	39.19	47.18	51.05	48.49	53.62	50.44	48.24	52.64
Female	50.54	47.55	53.53	56.86	52.82	60.81	48.95	46.38	51.51	49.56	47.36	51.76
Ethnicity %												
White	85.79	82.21	88.75	87.32	85.05	89.29	96.74	95.83	97.45	96.16	94.65	97.25
Black	5.15	3.49	7.55	2.13	1.38	3.27	1.35	0.88	2.06	1.45	0.78	2.67
Indian/Pakistani	4.93	3.29	7.32	4.85	3.75	6.26	1.18	0.78	1.78	1.60	0.92	2.74
Mixed race	1.24	0.43	3.48	2.37	1.71	3.27	0.44	0.25	0.76	0.34	0.19	0.61
Other/unsure	2.89	1.88	4.43	3.34	2.23	4.97	0.30	0.15	0.59	0.46	0.23	0.92
Relationship status, %												
Cohabiting relationship	6.88	5.56	8.48	65.40	61.81	68.82	68.76	66.06	71.34	67.55	65.32	69.70
Non-cohabiting relationship	33.75	30.72	36.93	11.73	9.77	14.04	10.63	9.06	12.44	13.39	11.85	15.10
Single	59.37	56.18	62.48	22.87	19.79	26.28	20.61	18.26	23.17	19.07	17.26	21.01
COVID-19 status, %												
Yes, confirmed	0.32	0.13	0.80	0.57	0.29	1.13	0.68	0.24	1.90	0.33	0.20	0.53
Yes, unconfirmed	5.17	4.11	6.48	10.26	8.08	12.95	9.54	7.94	11.41	5.42	4.62	6.35
Unsure	21.31	18.73	24.14	23.57	20.71	26.69	25.44	23.18	27.85	19.91	18.28	21.65
No	73.20	70.33	75.89	65.60	62.19	68.86	64.34	61.71	66.88	74.34	72.47	76.13
Economic activity, %												
Employed	62.61	57.17	67.75	80.82	77.42	83.81	69.34	66.61	71.93	44.05	41.85	46.27
Self-employed	2.43	1.41	4.17	6.32	4.67	8.49	12.81	11.36	14.43	12.13	10.52	13.93
Unpaid/voluntary work	0.11	0.02	0.48	0.21	0.09	0.52	0.14	0.07	0.28	0.48	0.31	0.73
Apprenticeship	6.16	4.32	8.73	0.11	0.03	0.38	-	-	-	-	-	-
Unemployed	20.40	15.79	25.94	4.10	2.82	5.92	3.96	2.69	5.78	3.56	2.65	4.78
Permanently sick or disabled	0.44	0.17	1.10	0.75	0.37	1.51	6.02	4.33	8.33	5.59	4.34	7.17
Looking after home or family	1.13	0.49	2.59	4.27	2.83	6.39	5.08	4.05	6.35	4.58	3.89	5.39
In education	1.43	0.67	3.03	-	-	-	0.03	0.01	0.15	-	-	-
Retired	-	-	-	-	-	-	1.02	0.61	1.70	28.04	26.29	29.86
Uncategorised	5.29	3.39	8.16	3.43	2.26	5.19	1.61	0.91	2.81	1.56	0.86	2.82
Key worker, %												
Yes	9.36	7.50	11.61	33.57	30.03	37.29	31.63	29.45	33.89	18.88	17.30	20.57
No	90.64	88.39	92.50	66.43	62.71	69.97	68.37	66.11	70.55	81.12	79.43	82.70
NS-SEC analytical classes, %												
Higher managerial	0.78	0.45	1.34	16.80	14.14	19.84	15.83	14.10	17.72	7.31	6.27	8.51
Lower managerial	3.05	1.90	4.86	29.71	26.35	33.30	20.48	18.73	22.35	12.23	10.87	13.73
Intermediate occupations	5.56	4.48	6.88	17.46	14.61	20.74	13.42	12.11	14.83	9.60	8.61	10.69
Small employers and self-employed	1.09	0.68	1.75	2.80	1.86	4.20	5.19	4.21	6.38	4.48	3.57	5.62
Lower supervisory and technical	2.39	1.55	3.68	3.19	1.86	5.43	4.87	3.94	5.99	3.84	3.04	4.86
Semi-routine occupations	11.35	9.29	13.81	9.23	7.35	11.53	9.66	8.35	11.14	9.59	8.27	11.09
Routine occupations	5.65	4.50	7.07	3.71	2.56	5.35	7.44	6.13	9.00	6.03	5.07	7.16
Uncategorised	70.13	66.38	73.62	17.09	14.56	19.96	23.12	20.56	25.91	46.92	44.72	49.12
Change in drinking, %												
Less	49.45	46.00	52.90	21.03	18.04	24.37	11.52	9.96	13.30	16.29	14.55	18.18
Same	36.43	33.34	39.63	49.89	45.93	53.85	61.81	59.28	64.28	65.43	63.25	67.54
More	14.12	11.49	17.24	29.08	25.65	32.77	26.67	24.49	28.96	18.28	16.78	19.89
Risk of alcohol-related harm at time of survey, %												
Low risk	77.87	74.33	81.04	70.68	66.79	74.29	59.99	57.48	62.45	62.29	60.16	64.38
Increasing risk	17.29	14.76	20.15	23.65	20.36	27.30	32.25	30.01	34.58	31.55	29.62	33.55
High risk	1.49	0.90	2.46	2.10	1.16	3.78	2.74	2.21	3.39	2.63	2.15	3.22
Highest risk	3.35	2.04	5.47	3.56	2.20	5.72	5.02	3.85	6.51	3.52	2.73	4.53
Change in stress, %												
Less	17.86	15.19	20.88	9.94	7.84	12.52	10.86	9.40	12.52	7.13	6.18	8.22
Same	44.97	41.73	48.25	45.04	41.32	48.82	50.28	47.68	52.88	60.64	58.49	62.75
More	37.17	34.37	40.06	45.02	41.30	48.80	38.85	36.33	41.44	32.23	30.25	34.28
Risk-taking, M (SD)	7.01 (2.18)	6.86	7.15	6.64 (2.22)	6.48	6.80	5.99 (2.53)	5.84	6.14	5.91 (2.64)	5.78	6.04
Impatience, M (SD)	4.30 (2.60)	4.14	4.46	4.27 (2.83)	4.04	4.51	4.03 (2.58)	3.89	4.17	3.88 (2.87)	3.73	4.03

Economic activity reflects activity during the pandemic

*MCS* Millennium Cohort Study, *BCS70* 1970 British Cohort Study, *NCDS* National Child Development Study, *NS-SEC* National Statistics Socio-economic class prior to the outbreak

### Outcome Measures

Alcohol use behaviour was assessed using five questions from the Alcohol Use Disorders Identification Test (AUDIT) — a tool developed by the World Health Organisation as a brief assessment of alcohol misuse (Babor et al., 2001). The original AUDIT has been shown to have excellent psychometric properties when used to assess alcohol use disorders in a variety of settings including both college students (Fleming et al., 1991) and during routine health examinations (Claussen & Aasland, 1993). Subsequently, several short versions of the AUDIT have been developed and shown to perform similarly to the original instrument (Gual, 2002; Kim et al., 2013).

The questions administered during the survey were as follows:

1. “How often have you had a drink containing alcohol?”
2. “How many standard alcoholic drinks have you had on a typical day when you were drinking?”
3. “How often have you found you were not able to stop drinking once you had started?”
4. “How often have you failed to do what was expected of you because of drinking?”
5. “Has a relative, friend, doctor, or health worker been concerned about your drinking or advised you to cut down?”

Questions one and two were repeated, prefaced by either “in the month before the Coronavirus outbreak”, or “since the start of the Coronavirus outbreak”. This provided an assessment of alcohol use prior to, and during, the pandemic. Questions 1 to 5 were posed in the context of the pandemic, and thus were worded using the latter phrasing, offering an assessment of hazardous drinking during the outbreak.

Each item was scored in line with the original AUDIT. Scores which represented alcohol use prior to and during the pandemic were calculated by summing questions one and two. A change score was calculated by subtracting the pre-pandemic from intra-pandemic score. Thus, values equal to zero reflected no change, values greater than zero represented an increase, and values less than zero denoted a reduction in alcohol use. A score representing risk of alcohol-related harm due to hazardous drinking during the pandemic was calculated by summing all items which used the latter wording. The hazardous drinking score was categorised proportionally to the original AUDIT. Whereby, a score between zero and three was coded as ‘Low risk’; a score between four and six was classified as ‘Increasing risk’; scores between seven and eight were labelled ‘Higher risk’; and scores of nine or greater were classed as ‘Highest risk’.

### Stress

Perceived stress was assessed using a single question: “Since the Coronavirus outbreak, please indicate how the following have changed… The amount of stress I’ve been feeling”. The possible responses included “More than before”, “Same — no change”, and “Less than before”. As it is well-known that experiencing symptoms of depression and/or anxiety is associated with increased psychological stress (Crawford & Henry, 2003; Heinen et al., 2017), we used linear regression models for each cohort to determine the relationship between scores on the Patient Health Questionnaire-4 (PHQ-4) (Kroenke et al., 2009) — an ultra-brief tool, with good psychometric properties, designed to screen for anxiety and depression in both clinical and non-clinical settings — and the stress item used here (see Supplementary Table 1). After controlling for potential confounders (see below), individuals who said they were feeling more stressed than before the pandemic scored approximately two points higher (range = 1.95–2.68, *p*_s_ < 0.001) than those who said they felt the same.

### Inhibitory Control

Two self-report measures of inhibitory control were administered in the survey: patience and risk-taking. Each was measured using a single ten-point Likert scale item. The questions were phrased “On a scale from 0 – 10, where 0 is ‘never’ and 10 is ‘always’, how *willing to take risks/patient* would say you are?”. A similar single-item scale of risk preference, known as the General Risk Question (GRQ) (Dohmen et al., 2011), has been used extensively and has been included in several widely analysed surveys, such as the Household, Income and Labour Dynamics in Australia Survey (Watson & Wooden, 2012), and the Understanding Society Survey (Institute for Social and Economic Research, 2018). Recent work suggests that the self-report (e.g., GRQ) assessment of risk-taking oftentimes outperforms behavioural assessments (e.g., laboratory lotteries) due to self-report assessments taking subjective internal states, such as regret or need, into account (Arslan et al., 2020). Moreover, during the development of the Global Preferences Survey (Falk et al., 2018) — which was conducted to investigate risk and time (patience) preferences — Falk et al. (Falk et al., 2016) experimentally validated their measures by (among other things) assessing the association between single-item assessments and behavioural measures of the same constructs through Spearman’s correlations and linear regression models. Their analysis shows that the single-item assessments were moderately correlated with the behavioural measures (see Supplementary Table 2). The “patience” item was reverse scored to reflect greater impatience.

### Potential Confounders

Potential confounding variables were identified using the authors’ substantive knowledge about established risk factors that could plausibly be related to our outcome variables. These included respondents’ sex, ethnicity, National Statistics Socio-economic Class (NS-SEC) prior to the outbreak of Coronavirus, and economic activity during the pandemic. Further information on these measures is presented in the Supplementary Material.

### Statistical Analysis

Statistical analysis was conducted using Stata IC (version 16.1). Figures were generated using *ggplot2* (version 3.3.2) for R (version 3.6.2). Inverse probability weighting was used to account for bias introduced due to missing data, and to ensure the results were as representative as possible (Seaman & White, 2013). The overall percentage of missing data was 23.43%. The median percentage of missing data by variable was 5.29% (IQR = 8.01%). See Supplementary Table 3 for a detailed description of missing data. Separate analyses were conducted for each cohort due to differences in sampling methods and therefore design weights. Descriptive statistics (mean and standard deviation or proportion alongside 95% CIs) were calculated for our variables of interest and selected the demographic variables. Prevalence estimates (with 95% CIs) split by sex, ethnicity, economic activity, and NS-SEC were calculated for our outcome measures and change in stress. Ordinal regression models were used to assess whether sociodemographic sub-group membership was associated with change in alcohol use, risk of alcohol-related harm due to hazardous drinking, or a change in stress levels, and to investigate associations between inhibitory control, stress, and alcohol use. We first regressed our outcome measures and change in stress on sex, ethnicity, economic activity, and NS-SEC. We then added parameters for inhibitory control, stress, and the interaction between inhibitory control and stress to our models containing our outcome variables. Given that most respondents across all cohorts were White, and since some ethnic groups made up less than 1% of the sample, a dichotomous White/non-White variable was used in regression analyses. We also noticed that the standard error among 50-year-olds that reported being in education during the pandemic was inflated, leading to implausible results, due to only two 50-year-old females falling into this category. These two cases were omitted for all regression analyses, which had no impact on the final results of the models. For brevity, model estimates for potential confounders are reported in the Supplementary Tables 4–15. Finally, as neither the study nor analysis plan was pre-registered on a publicly available platform, the results should be considered exploratory.

## Results

### Change in Alcohol Use During First Lockdown

Across all cohorts, most respondents reported drinking the same amount of alcohol or less since the start of the pandemic (Table 1). Thirty-year-olds and 50-year-olds were most likely to report increased drinking with around one-third and one-quarter reporting an increase respectively.

**Fig. 1:**
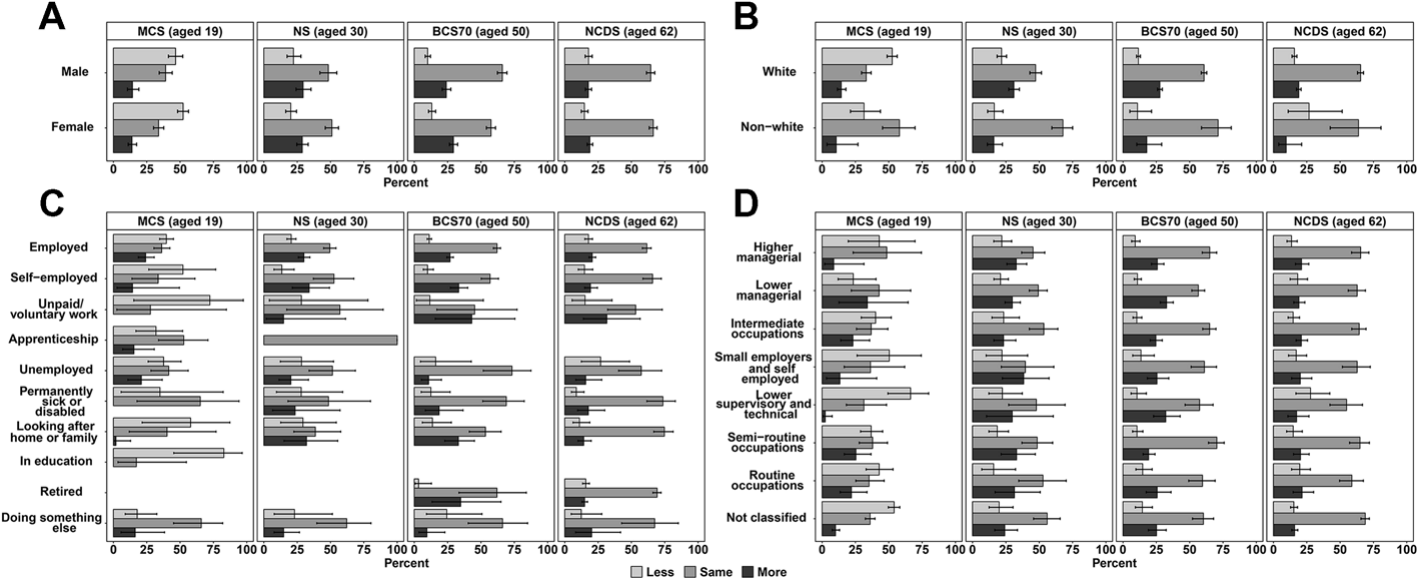
Change in alcohol use during the first wave (May 2020) of the COVID-19 pandemic in the UK, utilising data from four birth cohorts: The Millennium Cohort Study (*n* = 2,645), Next Steps (*n* = 1907), the 1970 British Cohort Study (*n* = 4223), and the National Child Development Study (*n* = 5178) by sex (**A**), ethnicity (**B**), economic activity during the pandemic (**C**), and National Statistics Socio-economic Class (**D**). Point estimates represent weighted percentages; error bars represent 95% confidence intervals

Figure 1 shows change in alcohol use by sub-group. In all cohorts except for 60-two-year-olds, being employed was associated with reporting increased alcohol use (Supplementary Tables 4–6). Fifty-year-old and 62-year-old females had 1.27 (95% CI 1.08 to 1.50) and 1.23 (95% CI 1.02 to 1.50) times the odds of reporting increased alcohol use, respectively (Supplementary Tables 6 and 7). Regarding socio-economic class, 50-year-olds who worked in intermediate occupations (OR = 0.70, 95% CI 0.54 to 0.92), semi-routine occupations (OR = 0.62, 95% CI 0.46 to 0.85), and routine occupations (OR = 0.62, 95% CI 0.39 to 0.98), and 62-year-olds in lower supervisory and technical occupations (OR = 0.45, 95% CI 0.24 to 0.84) were less likely to report an increase in alcohol use compared to those in higher managerial positions (Supplementary Tables 6 and 7). Finally, among 30-year-olds, non-White ethnicity was associated with a 29% (OR = 0.71, 95% CI 0.55 to 0.93) reduction in the odds of reporting increased drinking (Supplementary Table 4).

### Risk of Alcohol-Related Harm due to Hazardous Drinking During the First Lockdown

**Fig. 2:**
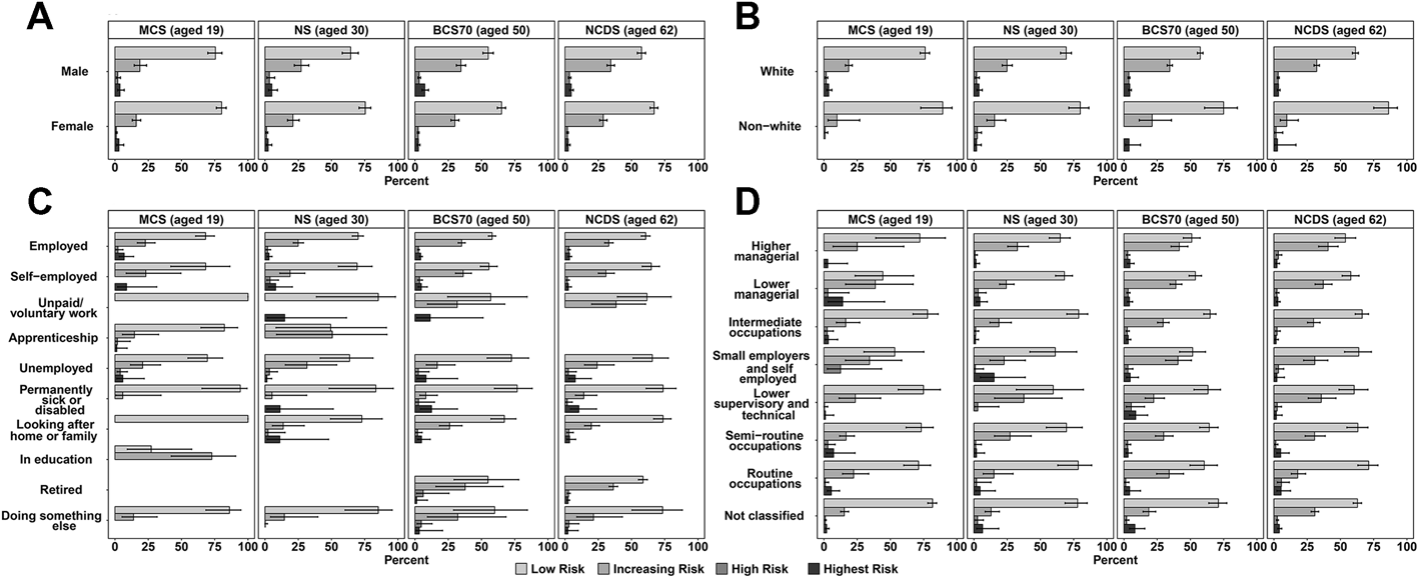
Risk of alcohol-related harm due to hazardous drinking during the first wave (May 2020) of the COVID-19 pandemic in the UK, utilising data from four birth cohorts: The Millennium Cohort Study (*n* = 2645), Next Steps (*n* = 1907), the 1970 British Cohort Study (*n* = 4,223), and the National Child Development Study (*n* = 5178) by sex (**A**), ethnicity (**B**), economic activity during the pandemic (**C**), and National Statistics Socio-economic Class (**D**). Point estimates represent weighted percentages; error bars represent 95% confidence intervals

Most participants fell into the low-risk category regardless of age or sub-group membership since the start of the lockdown (Table 1; Fig. 2). Approximately one-fifth of 19-year-olds, one-third of 30-year-olds, and two-fifths of both 50-year-olds and 62-year-olds were at an increased risk of alcohol-related harm or worse. Of these, approximately 60.50% (95% CI 48.73 to 71.17) of 19-year-olds, 59.93% (95% CI 52.51 to 66.92) of 30-year-olds, 68.11% (95% CI 63.14 to 72.71) of 50-year-olds, and 69.28% (95% CI 49.96, 73.29) of 62-year-olds reported an increase in alcohol use since the start of the pandemic.

Figure 2 shows risk of alcohol-related harm due to hazardous drinking by sub-group. Among 19-year-olds, being employed or in education was associated with an increase in the odds of being more at risk of alcohol-related harm (Supplementary Table 8). For 30-, 50-, and 62-year-olds (Supplementary Tables 9–11), being female and non-White ethnicity were associated with decreased odds of alcohol-related harm due to hazardous drinking. Finally, some effects were cohort specific. Being a permanently sick or disabled 50-year-old was associated with a 76% (OR = 0.24, 95% CI 0.10 to 0.58) decrease in the odds of alcohol related harm compared to those who were employed (Supplementary Table 10). Similarly, 62-year-olds who worked in routine occupations (OR = 0.56, 95% CI 0.33 to 0.96) were less likely to drink hazardously (Supplementary Table 11).

### Change in Stress

**Fig. 3:**
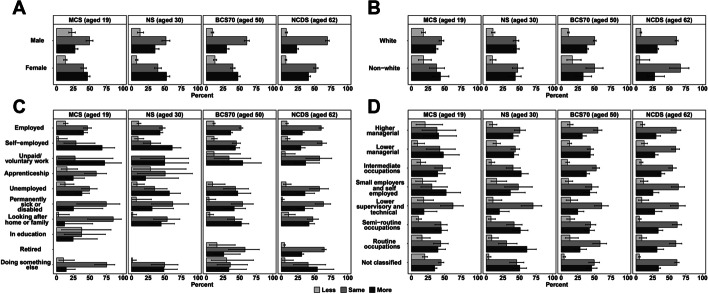
Change in perceived stress during the first wave (May 2020) of the COVID-19 pandemic in the UK, utilising data from four birth cohorts: the Millennium Cohort Study (*n* = 2645), Next Steps (*n* = 1907), the 1970 British Cohort Study (*n* = 4223), and the National Child Development Study (*n* = 5178) by sex (**A**), ethnicity (**B**), economic activity during the pandemic (**C**), and National Statistics Socio-economic Class (**D**). Point estimates represent weighted percentages; error bars represent 95% confidence intervals

Across all cohorts, most participants reported experiencing the same amount or less stress since the start of the pandemic (Table 1). Approximately two-fifths of 19-year-olds, half of 30-year-olds, two-fifths of 50-year-olds, and one-third of 62-year-olds reported feeling more stressed. Of those, females were disproportionately affected (Fig. 3). More specifically, among 19-year-olds, being female was associated with 1.54 (95% CI 1.08 to 2.20) times the odds of reporting an increase in stress (Supplementary Table 12). For 30-year-olds, being female was associated with 1.93 (95% CI 1.39 to 2.70) times the odds of reporting an increase in stress (Supplementary Table 13). For 50-year-olds, being female was associated with 1.62 (95% CI 1.37 to 1.92) times the odds of reporting an increase in stress (Supplementary Table 14). For 62-year-olds, being female was associated with 2.03 (95% CI 1.66 to 2.48) times the odds of reporting an increase in stress (Supplementary Table 15). Additionally, for 19-year-olds being either self-employed (OR = 5.53, 95% CI 1.56 to 19.57) or unemployed (OR = 1.75, 95% CI 1.08 to 2.83) was associated with an increase in the odds of reporting an increase in stress (Supplementary Table 12). Similarly, for 30-year-olds, being unemployed (OR = 2.14, 95% CI 1.15 to 3.98) was also associated with an increase in the odds of reporting an increase in stress (Supplementary Table 13).

### Associations Between Stress, Inhibitory Control, and Drinking Behaviour

#### Stress

After adjusting for potential confounders, 30-year-olds who reported feeling more stressed since the start of lockdown were at 3.77 (95% CI 1.15 to 12.28) times greater odds of being at increasing, high, or highest (versus low) risk of alcohol-related harm, compared to those that reported feeling no change in stress (Table 2). There was no evidence to suggest that this effect was present in other cohorts.

**Table 2:** Summary of the final ordinal regression models predicting change in drinking since the start of the pandemic (model A) and risk of alcohol-related harm due to hazardous drinking during the pandemic (model B), adjusting for sex, ethnicity, economic activity during the pandemic, and social class prior to the pandemic

	MCS			Next Steps			BCS70			NCDS		
Variable	OR (95% CI)	SE	*p*	OR (95% CI)	SE	*p*	OR (95% CI)	SE	*p*	OR (95% CI)	SE	*p*
**Model A: Change in drinking since the start of the pandemic**												
Stress												
Same	Ref			Ref			Ref			Ref		
Less	0.21 (0.02, 1.98)	0.24	0.172	0.59 (0.09, 3.71)	0.55	0.574	1.40 (0.58, 3.38)	0.63	0.455	1.38 (0.42, 4.51)	0.83	0.590
More	1.47 (0.39, 5.61)	1.00	0.568	2.21 (0.99, 4.94)	0.90	0.053	0.87 (0.51, 1.47)	0.23	0.594	0.90 (0.54, 1.48)	0.23	0.670
Risk-taking	0.98 (0.88, 1.10)	0.06	0.775	1.03 (0.95, 1.13)	0.05	0.479	0.98 (0.94, 1.03)	0.02	0.533	0.99 (0.94, 1.03)	0.02	0.508
Risk-taking × stress												
Same	Ref			Ref			Ref			Ref		
Less	1.2 (0.92, 1.57)	0.16	0.181	0.96 (0.74, 1.24)	0.13	0.760	0.96 (0.85, 1.08)	0.06	0.478	0.97 (0.82, 1.13)	0.08	0.674
More	1.05 (0.87, 1.26)	0.10	0.622	0.98 (0.87, 1.09)	0.06	0.676	1.06 (0.98, 1.15)	0.04	0.152	1.07 (0.98, 1.16)	0.04	0.118
Impatience	**1.14 (1.06, 1.24)**	**0.05**	**0.001**	1.05 (0.97, 1.14)	0.04	0.201	0.98 (0.93, 1.03)	0.02	0.370	0.99 (0.95, 1.03)	0.02	0.504
Impatience × stress												
Same	Ref			Ref			Ref			Ref		
Less	0.92 (0.75, 1.12)	0.09	0.404	**1.22 (1, 1.48)**	**0.12**	**0.047**	1.01 (0.9, 1.14)	0.06	0.846	1.01 (0.89, 1.15)	0.07	0.869
More	**0.87 (0.77, 0.99)**	**0.06**	**0.030**	**0.88 (0.8, 0.98)**	**0.05**	**0.016**	1.05 (0.97, 1.13)	0.04	0.216	0.99 (0.93, 1.07)	0.04	0.875
**Model B: Risk of alcohol-related harm due to hazardous drinking**												
Stress												
Same	Ref			Ref			Ref			Ref		
Less	0.25 (0.01, 4.98)	0.38	0.361	0.36 (0.06, 2.15)	0.33	0.259	1.00 (0.35, 2.89)	0.54	0.999	0.74 (0.25, 2.16)	0.41	0.585
More	0.82 (0.18, 3.65)	0.62	0.794	**3.77 (1.15, 12.28)**	**2.27**	**0.028**	1.29 (0.73, 2.25)	0.37	0.380	0.88 (0.49, 1.60)	0.27	0.680
Risk-taking	0.98 (0.8, 1.19)	0.10	0.836	**1.18 (1.05, 1.32)**	**0.07**	**0.006**	**1.06 (1.01, 1.12)**	**0.03**	**0.017**	1.00 (0.95, 1.05)	0.03	0.945
Risk-taking × stress												
Same	Ref			Ref			Ref			Ref		
Less	1.32 (0.89, 1.96)	0.27	0.172	0.97 (0.77, 1.22)	0.11	0.813	0.95 (0.82, 1.1)	0.07	0.504	1.04 (0.89, 1.22)	0.08	0.631
More	1.13 (0.91, 1.41)	0.12	0.258	0.88 (0.77, 1.02)	0.07	0.098	1.01 (0.93, 1.09)	0.04	0.834	1.08 (0.99, 1.18)	0.05	0.091
Impatience	**1.20 (1.05, 1.38)**	**0.08**	**0.010**	0.97 (0.9, 1.06)	0.04	0.531	1.00 (0.95, 1.04)	0.02	0.859	1.02 (0.97, 1.06)	0.02	0.480
Impatience × stress												
Same	Ref			Ref			Ref			Ref		
Less	0.9 (0.73, 1.12)	0.10	0.359	**1.31 (1.1, 1.57)**	**0.12**	**0.002**	**1.17 (1.04, 1.31)**	**0.07**	**0.007**	1.04 (0.94, 1.16)	0.06	0.435
More	0.95 (0.8, 1.14)	0.09	0.603	0.95 (0.83, 1.07)	0.06	0.396	1.00 (0.93, 1.08)	0.04	0.943	1.00 (0.92, 1.09)	0.04	0.972

Significant effects (*p* < .05) are in boldface

*MCS* Millennium Cohort Study, *BCS70* 1970 British Cohort Study, *NCDS* National Child Development Study. *NS-SEC* National Statistics Socio-economic class prior to the outbreak

#### Impatience

Among 19-year-olds, a one-unit increase in impatience was associated with 1.14 (95% CI 1.06 to 1.24) times the odds of reporting an increase in alcohol use, and 1.20 (OR = 1.20, 95% CI 1.05 to 1.38) times the odds of alcohol-related harm due to hazardous drinking after controlling for potential confounders (Table 2). There was no evidence to suggest that this effect was present in other cohorts.

#### Risk-Taking

After controlling for potential confounders, a one-unit increase in risk-taking was associated with 1.18 (95% CI 1.05 to 1.32) times the odds of alcohol-related harm among 30-year-olds (Table 2). Similarly, for 50-year-olds, a one-unit increase in risk-taking was associated with 1.06 (95% CI 1.01 to 1.12) times the odds of alcohol-related harm. This effect was not observed in other cohorts (Table 2).

#### Stress × Personality Interactions

There was evidence to suggest that, after controlling for potential confounders, individuals who were more impatient and *less stressed* tended to drink more and be at a greater risk of alcohol-related harm (Table 2). Specifically, for 30-year-olds, a one-unit increase in impatience was associated with a 22% (OR = 1.22, 95% CI 1.00 to 1.48) increase in the odds of reporting an increase in alcohol use among those who reported feeling less stressed, and a 12% (OR = 0.88, 95% CI 0.80 to 0.98) decrease in the odds of reporting an increase in alcohol use among those who reported feeling more stressed. Similarly, among 19-year-olds that reported feeling more stressed, a one-unit increase in impatience was associated with a 13% (OR = 0.87, 95% CI 0.77 to 0.99) decrease in the odds of reporting an increase in alcohol use. In terms of risk of alcohol-related harm, for both 30-year-olds (OR = 1.31, 95% CI 1.10 to 1.57) and 50-year-olds (OR = 1.17, 95% CI 1.04 to 1.31), reporting feeling less stressed was associated with an increase in the odds of being at an increased risk of alcohol-related harm or worse. No stress × personality interactions were observed in the 62-year-old cohort. No stress × risk-taking interactions were observed in any group.

## Discussion

The present study utilised data from four nationally representative British birth cohorts to explore changes in alcohol use behaviour and stress since the start of the COVID-19 outbreak, during the first national lockdown in May 2020. Across all age groups (cohorts), we found evidence to suggest that most respondents drank the same amount or less since the start of the pandemic. However, between approximately 14% and 30% of respondents reported drinking more depending on age. Of these, 30-year-olds and 50-year-olds were most likely to report an increase in drinking. This supports recent emerging evidence which suggests that between one-fifth and one-third of individuals in the UK reported drinking more during the first wave of the pandemic (Institute of Alcohol Studies, 2020; Jacob et al., 2021; Niedzwiedz et al., 2020). Further, between 20% and 40% of participants drank at levels of increasing risk of alcohol-related harm or worse, depending on age, with older participants displaying the greatest levels of risk due to alcohol misuse. Of these, approximately 60% of both 19-year-olds and 30-year-olds, and 70% of both 50-year-olds and 62-year-olds reported drinking more since the start of the pandemic. Provisional data from the Office for National Statistics data suggests that alcohol-related deaths reached a 20-year high between quarter one (January to March) and quarter three (July to September) of 2020; with significant increases in mortality among those aged between 30 and 49 in quarter 2 and 40 to 69 in quarter 3 (Office for National Statistics, 2021). These data add concerning weight to our findings of the higher rates of harmful drinking in these age groups, supporting the public health concerns attributable to excess alcohol use in some at-risk individuals during lockdown (Clay & Parker, 2020; Finlay & Gilmore, 2020; Rehm et al., 2020). The increase in alcohol-related deaths could be, at least partly, attributable to changes in mental health service provision during the pandemic and therefore increased psychological distress on top of that directly associated with stay-at-home orders (Columb et al., 2020; Da et al., 2020).

Similar to changes in drinking behaviour, most participants reported experiencing the same amount or less stress since the start of the pandemic. Nevertheless, between approximately 30% and 45% of respondents reported an increase in their stress level. Of these, 30-year-olds seemed to be most affected as more respondents from this group reported increased stress compared to the other cohorts. This group also had the highest proportion of individuals that reported increased alcohol use and there was evidence of an association between stress and hazardous drinking here too. Analogous to this finding, previous research suggests that the Millennial generation struggle with stress management considerably more than previous generations (Bland et al., 2012). Similarly, recent data from the UK Household Longitudinal Survey (Etheridge & Spantig, 2020) suggests that young individuals have seen larger declines in well-being during the first lockdown. Surprisingly, despite the well-established link between substance use and stress (Jose et al., 2000; Ruisoto & Contador, 2019), a main effect of stress was not observed in any other group. However, in all cohorts, being female was associated with an increased likelihood of reporting heightened stress; an effect which has consistently been reported elsewhere (Etheridge & Spantig, 2020; Niedzwiedz et al., 2020; Stanton et al., 2020). This may be due to (for example) an increased risk of psychiatric symptoms prior to, and after, suffering with COVID-19; an increased risk of domestic violence; and a disproportionate responsibility for domestic tasks including caring for family members (Almeida et al., 2020). In terms of drinking, our results suggest that for the 50- and 62-year-olds cohorts, being female was associated with an approximate 25% increase in the odds of reporting an increased alcohol use. Interestingly, however, across all cohorts, except the 19-year-olds, being female was associated with around a 40% reduction in the odds of alcohol-related harm due to hazardous drinking.

Several sociodemographic characteristics were related to change in both stress and alcohol use behaviour. For instance, in all but the oldest cohorts, employment was related to reporting increased alcohol use, and in the youngest cohort, both being employed or in-education was associated with an increased likelihood of hazardous drinking and subsequent alcohol-related harm. Similarly, among 50-year-olds those in higher managerial positions were more likely to report increased alcohol use. Meanwhile, for those aged 62, higher managerial positions were associated with an increased risk of alcohol related harm due to hazardous drinking. As off-premises alcohol consumption has been classified as ‘essential’ by the UK government (Reynolds & Wilkinson, 2020), this association is likely related to the physical and financial availability of alcohol (Babor et al., 2010; Rehm et al., 2020). In other words, those that are employed and/or high earners will generally be able to (financially) afford to drink more. Regarding changes in stress, unemployment was related to an increased likelihood of reporting heightened stress among both 19- and 30-year-olds. Also, self-employed 19-year-olds were more likely to report increased stress. Again, this was most likely associated with financial stability. For instance, many people who rely on state welfare have been receiving Universal Credit which has been shown to be associated with psychological distress (Wickham et al., 2020), and recent research has shown that self-employed people have suffered a large and disproportionate reduction in income during the pandemic (Yue & Cowling, 2021). Finally, in all but the youngest cohorts, there was evidence to suggest that non-White ethnicity was associated with a decreased likelihood of alcohol-related harm due to hazardous drinking, and among 30-year-olds non-White ethnicity was associated with a decreased likelihood of reporting and increase in alcohol use. This was unsurprising considering that results from several papers suggest that being White is a risk-factor for alcohol use and misuse (Bécares et al., 2011; Rao et al., 2015; Twigg & Moon, 2013).

Self-reported inhibitory control, and in some cases, a complex interaction between stress and personality was related to alcohol use and hazardous drinking during the lockdown. For example, in 30- and 50-year-olds, risk-taking personality was associated with an increased propensity to consume more alcohol and to have higher hazardous drinking scores. This corresponds to a large volume of literature which associates poor inhibitory control with substance misuse (Belin et al., 2008; Bosker et al., 2017; Dalley & Ersche, 2019; Ersche et al., 2010; Khemiri et al., 2016; Kreek et al., 2005; Lee et al., 2019; Voon et al., 2020). Moreover, the majority of 19-year-olds reported drinking less since the start of the pandemic. This was unsurprising considering the recent evidence of the ‘devaluation of alcohol’ among Generation Z (Kraus et al., 2020). This finding may also have been driven by the closure of on-trade drinking locations since drinking at venues such as pubs and bars is more common among young people (Ally et al., 2016), and reduced exposure to environments related with alcohol consumption has been associated with a reduction in drinking among young individuals during the pandemic (Winstock et al., 2020). However, critically, for 19-year-olds, impatience was related to increased alcohol use and risk of alcohol-related harm due to hazardous drinking during the pandemic. This group also had the highest levels of impatience across all cohorts. Taken together, these findings raise a concern about the potential for adults who have poor inhibitory control to be at particular risk of an escalation of alcohol misuse following the pandemic situation.

It is clear from previous research that there is an interaction between stress and personality factors that influences drinking behaviour. For example, people who experience acute stress show increases in craving for, and consumption of, alcohol (Clay & Parker, 2018; Clay et al., 2018). Here, counterintuitively, we found that greater impatience and decreased stress was associated with increased alcohol use among 30-year-olds and an increased hazardous drinking among both thirty-year-olds and 50-year-olds. Similarly, among 19- and 30-year-olds, those that rated themselves as more impatient and experienced increased stress were less likely to report increased alcohol consumption. As ‘drinking to cope’ was a prominent feature related to alcohol use during lockdown in the USA (Rodriguez et al., 2020), it may also be the case here. For instance, individuals with poor inhibitory control tend to use alcohol as a method of dealing with stress (Fede et al., 2020; Hamilton et al., 2013). Therefore, these individuals may have reduced stress levels due to their reported increased alcohol use. Alternatively, as the physiological response to long-term (chronic) and short-term (acute) stress differs (Stephens & Wand, 2012), it may be that the interaction between inhibitory control and chronic stress also differs. Therefore, future research should endeavour to investigate the impact of the interaction between different types of stress and inhibitory control in the context of alcohol use.

### Limitations

We acknowledge several limitations in our study. First, the survey was designed to capture information across several domains other than those relevant here. Therefore, to mitigate known issues related to respondent burden (e.g., satisficing), brevity was prioritised, which inevitably resulted in less detail than may be ideal in some of the measures used. For instance, single-item measures were used to assess risk-taking, impatience, and stress which may fail adequately to capture the full scope of these constructs (i.e., these measures may suffer from reduced content validity). This increases the uncertainty surrounding estimates calculated using these measures. Therefore, the use of single-item measures may also inflate standard errors and risk for type II error. Some of this potential error is offset by our large sample size; however, we found some effects that were not statistically significant despite relatively large effect sizes (e.g., among 30-year-olds that reported increased stress, OR = 2.21, 95% CI 0.99 to 4.94). Second, there may be individual differences in the way each question was interpreted. For instance, feelings of stress are subjective and vary between individuals (Sommerfeldt et al., 2019). Therefore, while some may find the pandemic and related period of social isolation as extremely stressful, others will find lockdown less stressful than pre-pandemic life. This may offer another explanation for why some that reported poor inhibitory control and lower levels of stress also reported increased alcohol use. Third, there is no way to independently verify self-report drinking; it is well-known that people under-estimate their alcohol consumption when asked on questionnaires due to social desirability bias, and often a lack of detailed memory of drinking episodes (Northcote & Livingston, 2011). It may, therefore, be that our data under-represent the true extent of drinking during the pandemic. Fourth, as stress and alcohol use prior the pandemic were measured retrospectively, at the time of the survey, a ‘true’ baseline was not established, thus precluding the ability to infer causal relationships. Fifth, we realise that it is difficult to accurately assess determinants of change and these considerations informed our analysis. Therefore, we purposefully tried to avoid spurious findings by not adjusting for baseline measures in our models (Glymour et al., 2005). Finally, while the RRs were relatively low, as in comparable national COVID-19 web surveys (e.g., Institute for Social and Economic Research, 2020), the longitudinal nature of birth cohort data allows for attrition-related bias to be minimised using sample weights calculated by the CLS team (Brown et al., 2020). However, there is a possibility that unobserved predictors of missing data may still influence results.

## Conclusions

In conclusion, we aimed to explore factors that influenced changes in alcohol use behaviour during the first COVID-19 lockdown in the UK, particularly concentrating on self-report stress and personality characteristics (risk-taking and impatience). We found that although most respondents drank either the same amount or less than prior to the pandemic, a significant minority, particularly of 30- and 50-year olds, drank more, often in amounts which could be classified hazardous, thus increasing their risk of potential alcohol-related harm. We also found that increases in drinking hazardously were predicted by personality (risk-taking, impatience) and environment (stress), although this was age specific. When considered in combination with recent data on alcohol-related deaths in the UK during the first three quarters of 2020, our findings suggest that hazardous drinking in a minority was strongly influenced by the pandemic and propose that this may be influenced by a combination of stress and personality factors, but also likely due to the availability of alcohol and inaccessible mental health services. We suggest that in future lockdowns, the government and public health officials pay particular attention to at-risk individuals, in terms of service provision, and consider critically the ‘essential’ nature of off-premises alcohol sales.

## Supplementary Information

Supplementary file1 (PDF 768 KB)

## Data Availability

All data used in this study are available from the UK Data Service Website (https://ukdataservice.ac.uk/) under the “Safeguarded” data access policy.

## Code Availability

Stata code written to conduct the analyses reported here is available from https://osf.io/wf2rj/.
