# Supplementary material for: Associations Between Self-reported Inhibitory Control, Stress, and Alcohol (Mis)use During the First Wave of the COVID-19 Pandemic in the UK: a National Cross-sectional Study Utilising Data From Four Birth Cohorts
Source: Int J Ment Health Addict. 2021 Aug 3;21(1):350–71. doi: 10.1007/s11469-021-00599-8 (PMC8330475; doi:10.1007/s11469-021-00599-8)
Supplement: Supplementary file 1 — Supplementary file1 (PDF 768 KB) [file 11469_2021_599_MOESM1_ESM.pdf]

Supporting Material

Supplement to:

Associations between self-reported inhibitory control, stress, and alcohol (mis)use during the first wave of the COVID-19 pandemic in the UK: a national cross-sectional study utilising data from four birth cohorts

SUPPLEMENTARY METHODS **2**
Figure 1. Study sample overview. MCS = Millennium Cohort Study (born 2000 – 02); NSHD = MRC National Survey of Health and Development (born 1946); NCDS = 1958 National Child Development Study; BCS70 = 1970 British Cohort Study; the Next Steps cohort were born 1989 – 90. Opted out = Those who requested not to be contacted further via phone, email, or by clicking the "opt-out" button, which was included in the invitation email. Responded = Anyone who completed the first block of the questionnaire. Data from Brown et al., 2020 (Brown et al., 2020). *2*
Table 1. Linear regression models used to assess the association between stress and PHQ-4 score. *3*
Table 2. Associations between single-item assessments, and behavioural assessments of inhibitory control. *5*
POTENTIAL CONFOUNDERS 5
MISSING DATA 6
Table 3. Percentage of missing data by variable. *7*
SUPPLEMENTARY RESULTS **8**
CHANGE IN ALCOHOL USE 8
Table 4. Ordinal logistic regression results for the Millennium Cohort Study with change in alcohol use as the outcome. *8*
Table 5. Ordinal logistic regression results for the Next Steps cohort with change in alcohol use as the outcome. *10*
Table 6. Ordinal logistic regression results for the 1970 British Cohort Study with change in alcohol use as the outcome. *12*
Table 7. Ordinal logistic regression results for the National Child Development Study with change in alcohol use as the outcome. *14*
RISK OF ALCOHOL-RELATED HARM DUE TO HAZARDOUS DRINKING 16
Table 8. Ordinal logistic regression results for the Millennium Cohort Study with risk of alcohol-related harm due to hazardous drinking as the outcome. *16*
Table 9. Ordinal logistic regression results for the Next Steps cohort with risk of alcohol-related harm due to hazardous drinking as the outcome. *18*
Table 10. Ordinal logistic regression results for the 1970 British Cohort Study with risk of alcohol-related harm due to hazardous drinking as the outcome. *20*
Table 11. Ordinal logistic regression results for the National Child Development Study with risk of alcohol-related harm due to hazardous drinking as the outcome. *22*
CHANGE IN STRESS 24
Table 12. Ordinal logistic regression results for the Millennium Cohort Study with change in stress as the outcome. *24*
Table 13. Ordinal logistic regression results for the Next Steps cohort with change in stress as the outcome. *25*
Table 14. Ordinal logistic regression results for the 1970 British Cohort Study with change in stress as the outcome. *26*
Table 15. Ordinal logistic regression results for the National Child Development Study with change in stress as the outcome. *27*
REFERENCES **28**

Supplementary Methods

Figure 1. Study sample overview. MCS = Millennium Cohort Study (born 2000 – 02); NSHD = MRC National Survey of Health and Development (born 1946); NCDS = 1958 National Child Development Study; BCS70 = 1970 British Cohort Study; the Next Steps cohort were born 1989 – 90. Opted out = Those who requested not to be contacted further via phone, email, or by clicking the “opt-out” button, which was included in the invitation email. Responded = Anyone who completed the first block of the questionnaire. Data from Brown et al., 2020 **(Brown et al., 2020)**.

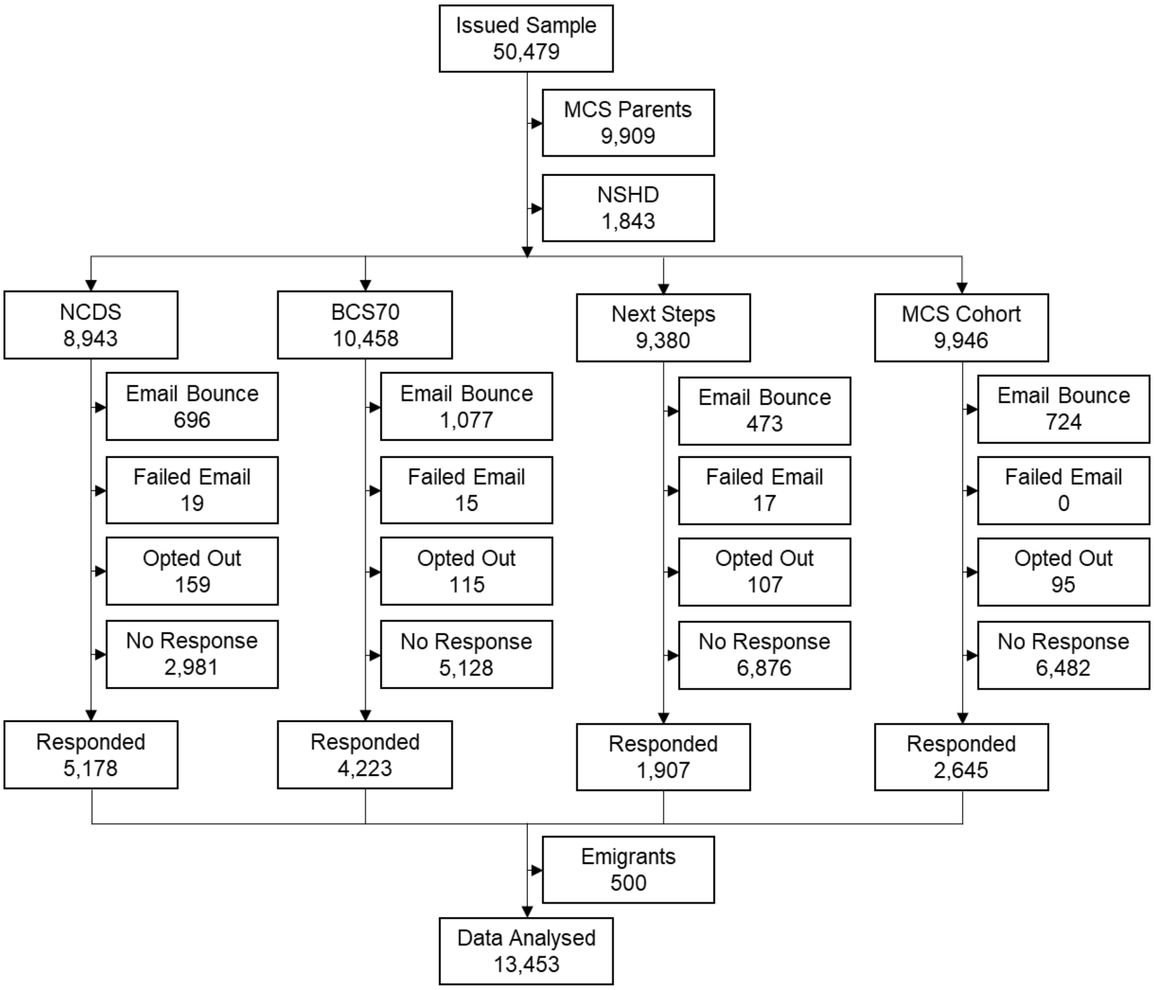

Table 1. Linear regression models used to assess the association between stress and PHQ-4 score.

	MCS						Next Steps						BCS70						NCDS					
**Variable**	**Coef.**	**LL**	**UL**	**SE**	***t***	***p***	**Coef.**	**LL**	**UL**	**SE**	***t***	***p***	**Coef.**	**LL**	**UL**	**SE**	***t***	***p***	**Coef.**	**LL**	**UL**	**SE**	***t***	***p***
**Unadjusted model**																								
Stress																								
Same - no change	Ref						Ref						Ref						Ref					
Less than before	-0.95	-1.40	-0.51	0.23	-4.18	**< .001**	-0.40	-0.80	-0.01	0.20	-2.00	**0.046**	0.20	-0.16	0.57	0.19	1.10	0.272	0.00	-0.28	0.29	0.15	0.02	0.983
More than before	2.45	2.02	2.88	0.22	11.21	**< .001**	2.60	2.17	3.04	0.22	11.72	**< .001**	2.16	1.78	2.54	0.19	11.09	**< .001**	2.16	1.89	2.44	0.14	15.48	**< .001**
**Adjusted model**																								
Stress																								
Same - no change	Ref						Ref						Ref						Ref					
Less than before	-0.41	-0.94	0.12	0.27	-1.52	0.13	-0.21	-0.62	0.19	0.20	-1.04	0.297	0.28	-0.03	0.59	0.16	1.76	0.078	0.06	-0.20	0.32	0.13	0.44	0.657
More than before	2.68	1.97	3.39	0.36	7.41	**< .001**	2.49	2.07	2.91	0.21	11.65	**< .001**	1.96	1.72	2.19	0.12	16.47	**< .001**	1.95	1.68	2.23	0.14	13.87	**< .001**
Sex																								
Male	Ref						Ref						Ref						Ref					
Female	0.68	0.05	1.32	0.32	2.13	**0.03**	0.50	0.09	0.91	0.21	2.42	**0.016**	0.01	-0.22	0.24	0.12	0.07	0.941	0.27	0.02	0.52	0.13	2.12	**0.034**
Ethnicity																								
White	Ref						Ref						Ref						Ref					
Non-white	0.04	-0.85	0.94	0.45	0.09	0.93	0.05	-0.37	0.47	0.21	0.24	0.809	-0.22	-0.58	0.13	0.18	-1.24	0.213	0.25	-0.31	0.81	0.29	0.87	0.385
NS-SEC 2010 analytical classes																								
Higher managerial	Ref						Ref						Ref						Ref					
Lower managerial	0.47	-1.39	2.33	0.94	0.50	0.62	-0.18	-0.73	0.37	0.28	-0.64	0.525	0.00	-0.25	0.25	0.13	0.03	0.976	0.08	-0.18	0.34	0.13	0.61	0.544
Intermediate occupations	0.28	-1.22	1.78	0.76	0.37	0.71	0.53	-0.23	1.30	0.39	1.38	0.169	0.31	0.02	0.60	0.15	2.08	**0.038**	0.19	-0.09	0.47	0.14	1.33	0.183

Small employer and self-employed	0.25	-1.98	2.48	1.13	0.22	0.83	0.43	-0.56	1.43	0.51	0.85	0.394	-0.17	-0.52	0.18	0.18	-0.95	0.343	0.18	-0.19	0.54	0.19	0.95	0.344
Lower supervisory and technical	0.04	-1.71	1.80	0.89	0.05	0.96	-0.37	-0.95	0.21	0.29	-1.25	0.212	0.57	-0.02	1.17	0.30	1.89	0.059	-0.11	-0.59	0.37	0.25	-0.45	0.655
Semi-routine occupations	0.38	-1.22	1.98	0.81	0.47	0.64	0.57	-0.24	1.39	0.41	1.38	0.169	0.53	0.10	0.95	0.22	2.42	0.016	0.49	0.04	0.94	0.23	2.15	**0.032**
Routine occupations	1.02	-0.67	2.71	0.86	1.19	0.24	-0.02	-1.13	1.09	0.56	-0.04	0.970	0.40	-0.07	0.87	0.24	1.67	0.095	0.41	-0.10	0.91	0.26	1.57	0.117
Uncategorised	1.30	-0.35	2.96	0.84	1.55	0.12	0.53	-0.59	1.65	0.57	0.93	0.352	0.46	0.03	0.88	0.22	2.11	**0.035**	0.38	-0.11	0.86	0.25	1.51	0.131
Economic activity																								
Employed	Ref						Ref						Ref						Ref					
Self-employed	-1.00	-3.04	1.04	1.04	-0.96	0.34	-0.37	-1.14	0.41	0.40	-0.92	0.356	-0.22	-0.47	0.03	0.13	-1.73	0.084	0.05	-0.31	0.41	0.18	0.26	0.795
In unpaid/voluntary work	2.02	-0.76	4.80	1.41	1.43	0.15	-0.41	-2.82	2.00	1.23	-0.33	0.738	-1.39	-1.95	-0.84	0.28	-4.90	**< .001**	-0.07	-1.21	1.07	0.58	-0.12	0.908
Apprenticeship	-0.39	-1.56	0.79	0.60	-0.65	0.52	0.44	-1.05	1.94	0.76	0.58	0.559	-	-	-	-	-	-	-	-	-	-	-	-
Unemployed	0.16	-0.73	1.04	0.45	0.35	0.73	1.39	-0.01	2.78	0.71	1.95	0.051	0.67	-0.22	1.56	0.45	1.47	0.141	0.26	-0.34	0.87	0.31	0.86	0.392
Permanently sick or disabled	2.86	-2.31	8.02	2.62	1.09	0.28	4.28	1.63	6.93	1.35	3.17	**0.002**	3.65	2.31	5.00	0.69	5.32	**< .001**	3.32	1.87	4.77	0.74	4.50	**< .001**
Looking after home or family	-1.30	-2.68	0.08	0.70	-1.86	0.07	0.90	-0.58	2.39	0.75	1.20	0.231	-0.03	-0.53	0.46	0.25	-0.13	0.894	-0.26	-0.86	0.35	0.31	-0.84	0.401
In education	0.34	-1.27	1.96	0.82	0.42	0.68	-	-	-	-	-	-	-	-	-	-	-	-	-	-	-	-	-	-
Retired	-	-	-	-	-	-	-	-	-	-	-	-	-0.61	-1.16	-0.06	0.28	-2.17	**0.030**	-0.25	-0.75	0.25	0.25	-0.98	0.326
Uncategorised	-1.12	-2.39	0.15	0.64	-1.73	0.08	-0.01	-1.26	1.25	0.64	-0.01	0.992	0.07	-1.36	1.49	0.73	0.09	0.925	0.46	-0.67	1.59	0.58	0.80	0.422

Note: NS-SEC = National Statistics Socio-economic class prior to the outbreak. Economic activity reflects activity during the pandemic.

Table 2. Associations between single-item assessments, and behavioural assessments of inhibitory control.

	Spearman's Correlation	OLS Coef.
Risk-taking item	0.35	0.20
Patience item	-0.40	-0.17

Note: Values represent the association between the single-item measures, and behavioural assessments, of risk-taking, and patience utilised in Falk et al., 2018(Falk et al., 2018). The Spearman's correlations were calculated using raw data, while the linear regression coefficients were calculated using standardised scores. N = 409. Adapted from Falk et al., 2016(Falk et al., 2016). OLS = ordinary least squares.

Potential confounders

Confounders included: respondent's sex (male or female); ethnicity (white or non-white); National Statistics Socioeconomic Class prior to the lockdown (NS-SEC, grouped into eight categories: higher managerial, lower managerial, intermediate occupations, small employers and self-employed, lower supervisory and technical, semi-routine occupations, routine occupations, and Uncategorised), and economic activity during the pandemic (grouped into ten categories: employed, self-employed, unpaid/voluntary work, apprenticeship, unemployed, permanently sick or disabled, looking after the home or family, in education, retired, and Uncategorised). The Office for National Statistics have published a detailed description of the NS-SEC (Office for National Statistics, 2016).

The selection of potential confounding variables was driven by the author's substantive knowledge about established risk factors that could plausibly be related to our outcome variables. For instance, there are several sociocultural factors that should be accounted for when researching alcohol misuse using human participants (Room, 2013). Historical data suggests that binge drinking is highest among younger individuals and declines with age (Office for National Statistics, 2018). However, recently emerging evidence suggests a devaluation of alcohol among Generation Z (born between 1996 and 2015) (Kraus et al., 2020). Similarly, in Western cultures men tend to drink more than woman, yet data from the US suggests a shift in the pattern, whereby rates of AUD have increased by around 85% among women (White et al., 2015). One explanation for this may be sex differences in susceptibility to stress (Peltier et al., 2019). In terms of ethnicity, binge drinking tends to be more prevalent among

white people (Twigg & Moon, 2013). This is thought to be partly attributable to the way alcohol consumption is often stigmatised among ethnic minorities (Room, 2005; Zapolski et al., 2014). Nevertheless, due to this stigmatisation, individuals that belong to these cultural groups tend to be disproportionately affected by alcohol-related harm (Zapolski et al., 2014). Further, having a lower socioeconomic status has been previously reported as being associated with lower total alcohol consumption, yet being at the greatest risk of hazardous drinking and alcohol-related harm, perhaps due to higher levels of heavy episodic (binge) drinking among more deprived groups (Mackenbach et al., 2008; Probst et al., 2020).

Missing data

Weights were derived from logistic regression models by the Centre for Longitudinal Studies team using several variables associated with non-response. For example, sex, ethnicity, social class, cognitive ability, indicators mental health, educational achievement, internet access prior to the web survey, economic activity, indicators of physical health, and non-response during previous sweeps – see Brown et al. (Brown et al., 2020) for a detailed description of the procedure used to calculate weights.

Table 3. Percentage of missing data by variable.

Variable	Overall	MCS	Next Steps	BCS70	NCDS
n	13,453	2,644	1,852	3,997	4,960
Sex	0.00%	0.00%	0.00%	0.00%	0.00%
Ethnicity	13.29%	3.82%	1.84%	10.56%	24.82%
Relationship status	2.70%	3.59%	2.92%	2.20%	2.54%
COVID-19 status	0.01%	0.00%	0.00%	0.03%	0.02%
Economic activity at time of survey	5.07%	8.17%	5.56%	3.53%	4.48%
Key worker	5.29%	8.28%	5.72%	0.00%	4.82%
NS–SEC 2010 analytical classes	0.00%	0.00%	0.00%	0.00%	0.00%
Change in drinking	7.87%	9.68%	6.70%	6.35%	8.57%
Alcohol misuse at time of survey	8.55%	10.25%	7.67%	6.96%	9.25%
Change in stress	8.07%	12.67%	9.67%	6.05%	6.65%
Risk-taking	7.83%	12.29%	8.96%	3.70%	6.43%
Impatience	8.02%	12.41%	8.96%	6.08%	6.88%
PHQ–4	0.00%	0.00%	0.00%	0.00%	0.00%

Note: NS-SEC = National Statistics Socio-economic Class; PHQ–4 = Patient Health Questionnaire – 4. The overall percentage of missing data was 23.43%.

Supplementary Results

Change in alcohol use

Table 4. Ordinal logistic regression results for the Millennium Cohort Study with change in alcohol use as the outcome.

	Model 1			Model 2		
**Variable**	**OR (95% CI)**	**SE**	***p***	**OR (95% CI)**	**SE**	***p***
Sex						
Male	Ref.			Ref.		
Female	0.77 (0.51, 1.14)	0.16	0.190	0.83 (0.55, 1.27)	0.18	0.400
Ethnicity						
White	Ref.			Ref.		
Non-white	1.39 (0.64, 3.01)	0.55	0.404	1.58 (0.72, 3.47)	0.63	0.251
NS-SEC analytical classes						
Higher managerial	Ref.			Ref.		
Lower managerial	3.77 (1.00, 14.23)	2.54	0.050	3.27 (0.81, 13.27)	2.33	0.096
Intermediate occupations	1.81 (0.68, 4.81)	0.90	0.235	1.84 (0.67, 5.07)	0.95	0.239
Small employers and self employed	1.11 (0.27, 4.54)	0.79	0.888	1.16 (0.25, 5.31)	0.90	0.851
Lower supervisory and technical	0.56 (0.18, 1.73)	0.32	0.309	0.56 (0.17, 1.85)	0.34	0.336
Semi-routine occupations	2.13 (0.83, 5.5)	1.03	0.118	2.1 (0.77, 5.77)	1.08	0.148
Routine occupations	1.93 (0.68, 5.43)	1.01	0.213	1.62 (0.55, 4.79)	0.89	0.380
Uncategorised	2.11 (0.72, 6.19)	1.15	0.173	2.13 (0.67, 6.8)	1.26	0.201
Economic activity						
Employed	Ref.			Ref.		
Self-employed	0.56 (0.15, 2.03)	0.37	0.375	0.55 (0.15, 2.05)	0.37	0.371
Unpaid/voluntary work	0.16 (0.02, 1.26)	0.17	0.081	0.16 (0.01, 1.74)	0.19	0.131
Apprenticeship	1.37 (0.71, 2.63)	0.45	0.343	1.38 (0.68, 2.79)	0.49	0.370
Unemployed	0.80 (0.42, 1.54)	0.27	0.505	0.70 (0.35, 1.41)	0.25	0.319
Permanently sick or disabled	0.71 (0.17, 2.96)	0.51	0.637	0.54 (0.13, 2.29)	0.40	0.405
Retired	-	-	-	-	-	-
Looking after home or family	0.70 (0.25, 1.97)	0.37	0.504	0.48 (0.17, 1.39)	0.26	0.177
In education	**0.10 (0.02, 0.59)**	**0.09**	**0.011**	**0.12 (0.03, 0.49)**	**0.09**	**0.004**
Uncategorised	0.95 (0.52, 1.73)	0.29	0.858	0.88 (0.44, 1.75)	0.31	0.709
Stress						
Same	Ref.			Ref.		

Less		0.21 (0.02, 1.98)	0.24	0.172
More		1.47 (0.39, 5.61)	1.00	0.568
Risk-taking		0.98 (0.88, 1.10)	0.06	0.775
Risk-taking x Stress				
Same	Ref.	Ref.		
Less		1.2 (0.92, 1.57)	0.16	0.181
More		1.05 (0.87, 1.26)	0.10	0.622
Impatience		**1.14 (1.06, 1.24)**	**0.05**	**0.001**
Impatience x Stress				
Same	Ref.	Ref.		
Less		0.92 (0.75, 1.12)	0.09	0.404
More		**0.87 (0.77, 0.99)**	**0.06**	**0.030**

NS-SEC = National Statistics Socio-economic Class. Model 1: Demographics (sex, ethnicity, NS-SEC prior to the outbreak of Coronavirus, and economic activity during the pandemic). Model 2: The effect of inhibitory control (risk-taking and patience), stress, and the interaction between inhibitory control and stress, adjusting for demographics.

Table 5. Ordinal logistic regression results for the Next Steps cohort with change in alcohol use as the outcome.

	Model 1			Model 2		
**Variable**	**OR (95% CI)**	**SE**	***p***	**OR (95% CI)**	**SE**	***p***
Sex						
Male	Ref.			Ref.		
Female	1.14 (0.85, 1.54)	0.17	0.380	1.17 (0.86, 1.59)	0.18	0.315
Ethnicity						
White	Ref.			Ref.		
Non-white	**0.71 (0.55, 0.93)**	**0.10**	**0.012**	**0.70 (0.54, 0.91)**	**0.09**	**0.008**
NS-SEC analytical classes						
Higher managerial	Ref.			Ref.		
Lower managerial	0.90 (0.61, 1.32)	0.18	0.575	0.91 (0.62, 1.34)	0.18	0.640
Intermediate occupations	0.72 (0.45, 1.14)	0.17	0.161	0.64 (0.39, 1.05)	0.16	0.075
Small employers and self employed	0.96 (0.39, 2.39)	0.44	0.937	0.88 (0.35, 2.23)	0.42	0.788
Lower supervisory and technical	0.90 (0.31, 2.6)	0.49	0.850	0.91 (0.33, 2.5)	0.47	0.851
Semi-routine occupations	1.11 (0.63, 1.97)	0.32	0.713	1.19 (0.65, 2.15)	0.36	0.574
Routine occupations	1.01 (0.52, 1.96)	0.34	0.985	1.14 (0.51, 2.54)	0.46	0.743
Uncategorised	1.59 (0.81, 3.1)	0.54	0.175	1.36 (0.68, 2.7)	0.48	0.384
Economic activity						
Employed	Ref.			Ref.		
Self-employed	1.36 (0.82, 2.25)	0.35	0.235	1.22 (0.73, 2.04)	0.32	0.458
Unpaid/voluntary work	0.39 (0.05, 2.99)	0.40	0.363	0.47 (0.06, 3.62)	0.49	0.467
Apprenticeship	0.79 (0.54, 1.15)	0.15	0.212	0.89 (0.59, 1.33)	0.18	0.562
Unemployed	0.45 (0.19, 1.06)	0.20	0.069	0.51 (0.2, 1.29)	0.24	0.155
Permanently sick or disabled	0.39 (0.11, 1.35)	0.25	0.138	0.44 (0.12, 1.57)	0.29	0.207
Retired	-	-	-	-	-	-
Looking after home or family	0.47 (0.14, 1.54)	0.28	0.213	0.58 (0.17, 1.94)	0.36	0.377
In education	-	-	-	-	-	-
Uncategorised	**0.42 (0.17, 0.99)**	**0.19**	**0.049**	0.45 (0.18, 1.11)	0.21	0.083
Stress						
Same	Ref.			Ref.		
Less				0.59 (0.09, 3.71)	0.55	0.574
More				2.21 (0.99, 4.94)	0.90	0.053
Risk-taking				1.03 (0.95, 1.13)	0.05	0.479
Risk-taking x Stress						
Same	Ref.			Ref.		

Less		0.96 (0.74, 1.24)	0.13	0.760
More		0.98 (0.87, 1.09)	0.06	0.676
Impatience		1.05 (0.97, 1.14)	0.04	0.201
Impatience x Stress				
Same	Ref.	Ref.		
Less		**1.22 (1.00, 1.48)**	**0.12**	**0.047**
More		**0.88 (0.80, 0.98)**	**0.05**	**0.016**

NS-SEC = National Statistics Socio-economic Class. Model 1: Demographics (sex, ethnicity, NS-SEC prior to the outbreak of Coronavirus, and economic activity during the pandemic). Model 2: The effect of inhibitory control (risk-taking and patience), stress, and the interaction between inhibitory control and stress, adjusting for demographics.

Table 6. Ordinal logistic regression results for the 1970 British Cohort Study with change in alcohol use as the outcome.

	Model 1			Model 2		
**Variable**	**OR (95% CI)**	**SE**	***p***	**OR (95% CI)**	**SE**	***p***
Sex						
Male	Ref.			Ref.		
Female	**1.27 (1.08, 1.50)**	**0.11**	**0.004**	**1.19 (1.01, 1.41)**	**0.10**	**0.043**
Ethnicity						
White	Ref.			Ref.		
Non-white	0.77 (0.51, 1.16)	0.16	0.205	0.78 (0.51, 1.18)	0.16	0.234
NS-SEC analytical classes						
Higher managerial	Ref.			Ref.		
Lower managerial	0.96 (0.76, 1.22)	0.12	0.753	0.95 (0.75, 1.22)	0.12	0.708
Intermediate occupations	**0.70 (0.54, 0.92)**	**0.10**	**0.010**	**0.70 (0.53, 0.92)**	**0.10**	**0.010**
Small employers and self employed	0.78 (0.49, 1.23)	0.18	0.287	0.83 (0.52, 1.34)	0.20	0.444
Lower supervisory and technical	0.99 (0.65, 1.51)	0.21	0.955	1.02 (0.66, 1.58)	0.23	0.932
Semi-routine occupations	**0.62 (0.46, 0.85)**	**0.10**	**0.003**	**0.59 (0.43, 0.81)**	**0.10**	**0.001**
Routine occupations	**0.62 (0.39, 0.98)**	**0.15**	**0.041**	**0.56 (0.36, 0.87)**	**0.12**	**0.009**
Uncategorised	1.02 (0.68, 1.54)	0.21	0.912	1.17 (0.8, 1.71)	0.23	0.432
Economic activity						
Employed	Ref.			Ref.		
Self-employed	1.11 (0.85, 1.45)	0.15	0.435	0.98 (0.76, 1.25)	0.12	0.851
Unpaid/voluntary work	1.44 (0.33, 6.35)	1.09	0.633	1.37 (0.32, 5.94)	1.03	0.673
Apprenticeship	-	-	-	-	-	-
Unemployed	0.73 (0.43, 1.25)	0.20	0.253	0.66 (0.38, 1.15)	0.19	0.145
Permanently sick or disabled	**0.40 (0.24, 0.66)**	**0.10**	**< 0.001**	**0.35 (0.21, 0.56)**	**0.09**	**< 0.001**
Retired	1.09 (0.67, 1.75)	0.27	0.732	0.97 (0.61, 1.55)	0.23	0.900
Looking after home or family	0.87 (0.47, 1.6)	0.27	0.655	0.91 (0.49, 1.7)	0.29	0.766
In education	-	-	-	-	-	-
Uncategorised	**0.36 (0.14, 0.94)**	**0.18**	**0.036**	**0.33 (0.12, 0.9)**	**0.17**	**0.031**
Stress						
Same	Ref.			Ref.		
Less				1.40 (0.58, 3.38)	0.63	0.455
More				0.87 (0.51, 1.47)	0.23	0.594
Risk-taking				0.98 (0.94, 1.03)	0.02	0.533
Risk-taking x Stress						

Same	Ref.	Ref.		
Less		0.96 (0.85, 1.08)	0.06	0.478
More		1.06 (0.98, 1.15)	0.04	0.152
Impatience		0.98 (0.93, 1.03)	0.02	0.370
Impatience x Stress				
Same	Ref.	Ref.		
Less		1.01 (0.9, 1.14)	0.06	0.846
More		1.05 (0.97, 1.13)	0.04	0.216

NS-SEC = National Statistics Socio-economic Class. Model 1: Demographics (sex, ethnicity, NS-SEC prior to the outbreak of Coronavirus, and economic activity during the pandemic). Model 2: The effect of inhibitory control (risk-taking and patience), stress, and the interaction between inhibitory control and stress, adjusting for demographics.

Table 7. Ordinal logistic regression results for the National Child Development Study with change in alcohol use as the outcome.

	Model 1			Model 2		
**Variable**	**OR (95% CI)**	**SE**	***p***	**OR (95% CI)**	**SE**	***p***
Sex						
Male	Ref.			Ref.		
Female	**1.23 (1.02, 1.50)**	**0.12**	**0.035**	1.19 (0.98, 1.44)	0.12	0.081
Ethnicity						
White	Ref.			Ref.		
Non-white	0.54 (0.26, 1.09)	0.19	0.083	0.53 (0.26, 1.08)	0.19	0.081
NS-SEC analytical classes						
Higher managerial	Ref.			Ref.		
Lower managerial	0.85 (0.6, 1.2)	0.15	0.354	0.88 (0.61, 1.25)	0.16	0.467
Intermediate occupations	0.9 (0.62, 1.31)	0.17	0.593	0.91 (0.62, 1.32)	0.17	0.610
Small employers and self employed	0.73 (0.44, 1.22)	0.19	0.235	0.81 (0.5, 1.34)	0.21	0.418
Lower supervisory and technical	**0.45 (0.24, 0.84)**	**0.14**	**0.012**	**0.45 (0.24, 0.86)**	**0.15**	**0.015**
Semi-routine occupations	0.96 (0.59, 1.57)	0.24	0.870	1.03 (0.63, 1.69)	0.26	0.896
Routine occupations	0.76 (0.48, 1.22)	0.18	0.260	0.79 (0.49, 1.28)	0.19	0.334
Uncategorised	0.7 (0.4, 1.22)	0.20	0.206	0.71 (0.39, 1.28)	0.21	0.257
Economic activity						
Employed	Ref.			Ref.		
Self-employed	0.94 (0.7, 1.26)	0.14	0.669	0.91 (0.66, 1.25)	0.15	0.566
Unpaid/voluntary work	1.61 (0.45, 5.71)	1.04	0.462	1.51 (0.42, 5.46)	0.99	0.529
Apprenticeship	-	-	-	-	-	-
Unemployed	0.69 (0.21, 2.22)	0.41	0.532	0.65 (0.21, 2)	0.37	0.453
Permanently sick or disabled	1.11 (0.61, 2.02)	0.34	0.743	1.13 (0.6, 2.13)	0.37	0.706
Retired	1.00 (0.59, 1.71)	0.27	0.988	1.01 (0.58, 1.75)	0.28	0.978
Looking after home or family	1.04 (0.55, 1.98)	0.34	0.903	1.05 (0.54, 2.06)	0.36	0.881
In education	-	-	-	-	-	-
Uncategorised	1.08 (0.32, 3.7)	0.68	0.902	1.04 (0.28, 3.91)	0.70	0.957
Stress						
Same	Ref.			Ref.		
Less				1.38 (0.42, 4.51)	0.83	0.590
More				0.90 (0.54, 1.48)	0.23	0.670
Risk-taking				0.99 (0.94, 1.03)	0.02	0.508
Risk-taking x Stress						
Same	Ref.			Ref.		

Less		0.97 (0.82, 1.13)	0.08	0.674
More		1.07 (0.98, 1.16)	0.04	0.118
Impatience		0.99 (0.95, 1.03)	0.02	0.504
Impatience x Stress				
Same	Ref.	Ref.		
Less		1.01 (0.89, 1.15)	0.07	0.869
More		0.99 (0.93, 1.07)	0.04	0.875

NS-SEC = National Statistics Socio-economic Class. Model 1: Demographics (sex, ethnicity, NS-SEC prior to the outbreak of Coronavirus, and economic activity during the pandemic). Model 2: The effect of inhibitory control (risk-taking and patience), stress, and the interaction between inhibitory control and stress, adjusting for demographics.

Risk of alcohol-related harm due to hazardous drinking

Table 8. Ordinal logistic regression results for the Millennium Cohort Study with risk of alcohol-related harm due to hazardous drinking as the outcome.

	Model 1			Model 2		
**Variable**	**OR (95% CI)**	**SE**	***p***	**OR (95% CI)**	**SE**	***p***
Sex						
Male	Ref.			Ref.		
Female	0.60 (0.36, 1.02)	0.16	0.062	0.58 (0.34, 1.02)	0.16	0.057
Ethnicity						
White	Ref.			Ref.		
Non-white	0.55 (0.23, 1.33)	0.25	0.185	0.71 (0.3, 1.67)	0.31	0.427
NS-SEC analytical classes						
Higher managerial	Ref.			Ref.		
Lower managerial	1.76 (0.39, 8.04)	1.36	0.463	1.47 (0.28, 7.77)	1.24	0.649
Intermediate occupations	0.67 (0.17, 2.59)	0.46	0.557	0.71 (0.16, 3.15)	0.54	0.655
Small employers and self employed	1.46 (0.33, 6.5)	1.11	0.618	1.04 (0.21, 5.14)	0.84	0.963
Lower supervisory and technical	0.34 (0.08, 1.53)	0.26	0.159	0.29 (0.06, 1.48)	0.24	0.135
Semi-routine occupations	0.74 (0.18, 3.09)	0.54	0.681	0.69 (0.14, 3.36)	0.56	0.647
Routine occupations	0.68 (0.17, 2.75)	0.48	0.588	0.56 (0.12, 2.71)	0.45	0.472
Uncategorised	1.08 (0.27, 4.33)	0.76	0.917	1.36 (0.3, 6.21)	1.05	0.687
Economic activity						
Employed	Ref.			Ref.		
Self-employed	0.84 (0.27, 2.58)	0.48	0.757	0.65 (0.2, 2.17)	0.40	0.485
Unpaid/voluntary work	**5.60E-07 (1.30E-07, 2.42E-06)**	**4.16E-07**	**< 0.001**	**6.40E-07 (1.04E-07, 3.96E-06)**	**5.92E-07**	**< 0.001**
Apprenticeship	**0.29 (0.11, 0.78)**	**0.15**	**0.015**	**0.25 (0.08, 0.77)**	**0.14**	**0.016**
Unemployed	0.79 (0.39, 1.61)	0.29	0.521	0.72 (0.37, 1.4)	0.24	0.333
Permanently sick or disabled	**0.11 (0.01, 1.03)**	**0.12**	**0.053**	**0.07 (0.01, 0.68)**	**0.08**	**0.021**
Retired	-	-	-	-	-	-
Looking after home or family	**6.91E-07 (2.37E-07, 2.01E-06)**	**3.75E-07,**	**< 0.001**	**5.86E-07 (9.92E-08, 3.46E-06)**	**5.29E-07**	**< 0.001**
In education	**2.63 (1.37, 5.05)**	**0.87**	**0.004**	2.16 (0.93, 5.04)	0.93	0.075
Uncategorised	**0.18 (0.05, 0.62)**	**0.11**	**0.007**	**0.11 (0.03, 0.46)**	**0.08**	**0.003**

Stress				
Same	Ref.	Ref.		
Less		0.25 (0.01, 4.98)	0.38	0.361
More		0.82 (0.18, 3.65)	0.62	0.794
Risk-taking		0.98 (0.8, 1.19)	0.10	0.836
Risk-taking x Stress				
Same	Ref.	Ref.		
Less		1.32 (0.89, 1.96)	0.27	0.172
More		1.13 (0.91, 1.41)	0.12	0.258
Impatience		**1.20 (1.05, 1.38)**	**0.08**	**0.010**
Impatience x Stress				
Same	Ref.	Ref.		
Less		0.9 (0.73, 1.12)	0.10	0.359
More		0.95 (0.8, 1.14)	0.09	0.603

NS-SEC = National Statistics Socio-economic Class. Model 1: Demographics (sex, ethnicity, NS-SEC prior to the outbreak of Coronavirus, and economic activity during the pandemic). Model 2: The effect of inhibitory control (risk-taking and patience), stress, and the interaction between inhibitory control and stress, adjusting for demographics.

Table 9. Ordinal logistic regression results for the Next Steps cohort with risk of alcohol-related harm due to hazardous drinking as the outcome.

	Model 1			Model 2		
**Variable**	**OR (95% CI)**	**SE**	***p***	**OR (95% CI)**	**SE**	***p***
Sex						
Male	Ref.			Ref.		
Female	**0.60 (0.42, 0.85)**	**0.11**	**0.004**	**0.56 (0.39, 0.82)**	**0.11**	**0.003**
Ethnicity						
White	Ref.			Ref.		
Non-white	**0.55 (0.34, 0.91)**	**0.14**	**0.02**	**0.44 (0.27, 0.73)**	**0.11**	**0.002**
NS-SEC analytical classes						
Higher managerial	Ref.			Ref.		
Lower managerial	1.03 (0.65, 1.63)	0.24	0.893	1.02 (0.65, 1.6)	0.24	0.935
Intermediate occupations	0.6 (0.37, 0.97)	0.15	0.037	0.59 (0.35, 0.99)	0.16	0.047
Small employers and self employed	1.43 (0.57, 3.58)	0.67	0.448	1.2 (0.41, 3.52)	0.66	0.741
Lower supervisory and technical	1.03 (0.36, 2.93)	0.55	0.956	1.00 (0.35, 2.87)	0.54	0.998
Semi-routine occupations	0.99 (0.49, 1.97)	0.35	0.971	0.93 (0.44, 1.94)	0.35	0.845
Routine occupations	0.55 (0.23, 1.32)	0.25	0.181	0.62 (0.24, 1.61)	0.3	0.327
Uncategorised	0.63 (0.23, 1.7)	0.32	0.357	0.6 (0.21, 1.69)	0.32	0.328
Economic activity						
Employed	Ref.			Ref.		
Self-employed	1.19 (0.64, 2.22)	0.38	0.584	1.12 (0.57, 2.2)	0.38	0.732
Unpaid/voluntary work	0.66 (0.03, 14.5)	1.04	0.793	0.64 (0.02, 19.07)	1.1	0.795
Apprenticeship	1.73 (0.41, 7.28)	1.27	0.454	2.53 (0.62, 10.32)	1.81	0.196
Unemployed	1.78 (0.56, 5.68)	1.05	0.327	2.20 (0.72, 6.74)	1.25	0.167
Permanently sick or disabled	0.68 (0.08, 5.73)	0.74	0.721	0.91 (0.11, 7.71)	0.99	0.928
Retired	-	-	-	-	-	-
Looking after home or family	1.73 (0.36, 8.33)	1.38	0.496	2.38 (0.44, 12.94)	2.05	0.315
In education	-	-	-	-	-	-
Uncategorised	0.64 (0.13, 3.24)	0.53	0.589	0.66 (0.13, 3.42)	0.55	0.617
Stress						
Same	Ref.			Ref.		
Less				0.36 (0.06, 2.15)	0.33	0.259
More				**3.77 (1.15, 12.28)**	**2.27**	**0.028**
Risk-taking				**1.18 (1.05, 1.32)**	**0.07**	**0.006**
Risk-taking x Stress						
Same	Ref.			Ref.		

Less		0.97 (0.77, 1.22)	0.11	0.813
More		0.88 (0.77, 1.02)	0.07	0.098
Impatience		0.97 (0.9, 1.06)	0.04	0.531
Impatience x Stress				
Same	Ref.	Ref.		
Less		**1.31 (1.10, 1.57)**	**0.12**	**0.002**
More		0.95 (0.83, 1.07)	0.06	0.396

NS-SEC = National Statistics Socio-economic Class. Model 1: Demographics (sex, ethnicity, NS-SEC prior to the outbreak of Coronavirus, and economic activity during the pandemic). Model 2: The effect of inhibitory control (risk-taking and patience), stress, and the interaction between inhibitory control and stress, adjusting for demographics.

Table 10. Ordinal logistic regression results for the 1970 British Cohort Study with risk of alcohol-related harm due to hazardous drinking as the outcome.

	Model 1			Model 2		
**Variable**	**OR (95% CI)**	**SE**	***p***	**OR (95% CI)**	**SE**	***p***
Sex						
Male	Ref.			Ref.		
Female	**0.64 (0.53, 0.76)**	**0.06**	**< 0.001**	**0.64 (0.53, 0.77)**	**0.06**	**< 0.001**
Ethnicity						
White	Ref.			Ref.		
Non-white	**0.44 (0.23, 0.84)**	**0.15**	**< 0.001**	**0.41 (0.21, 0.81)**	**0.14**	**0.010**
NS-SEC analytical classes						
Higher managerial	Ref.			Ref.		
Lower managerial	1.03 (0.83, 1.29)	0.12	0.775	1.03 (0.82, 1.29)	0.12	0.808
Intermediate occupations	0.79 (0.6, 1.04)	0.11	0.098	0.82 (0.62, 1.08)	0.12	0.164
Small employers and self employed	0.85 (0.55, 1.32)	0.19	0.478	0.87 (0.56, 1.36)	0.20	0.536
Lower supervisory and technical	0.70 (0.42, 1.18)	0.19	0.183	0.76 (0.45, 1.27)	0.20	0.289
Semi-routine occupations	0.85 (0.61, 1.18)	0.14	0.335	0.84 (0.6, 1.18)	0.15	0.321
Routine occupations	0.70 (0.44, 1.11)	0.17	0.132	**0.65 (0.44, 0.96)**	**0.13**	**0.030**
Uncategorised	0.91 (0.6, 1.38)	0.19	0.652	1.02 (0.68, 1.53)	0.21	0.939
Economic activity						
Employed	Ref.			Ref.		
Self-employed	1.1 (0.82, 1.48)	0.16	0.512	1.01 (0.77, 1.32)	0.14	0.944
Unpaid/voluntary work	1.12 (0.24, 5.14)	0.87	0.884	1.12 (0.24, 5.1)	0.87	0.886
Apprenticeship	-	-	-	-	-	-
Unemployed	0.83 (0.43, 1.6)	0.28	0.584	0.77 (0.41, 1.45)	0.25	0.422
Permanently sick or disabled	**0.24 (0.10, 0.58)**	**0.11**	**0.002**	**0.21 (0.09, 0.53)**	**0.10**	**0.001**
Retired	0.89 (0.54, 1.47)	0.23	0.653	0.82 (0.49, 1.36)	0.21	0.437
Looking after home or family	0.7 (0.26, 1.86)	0.35	0.475	0.72 (0.28, 1.85)	0.35	0.498
In education	-	-	-	-	-	-
Uncategorised	0.7 (0.28, 1.75)	0.33	0.442	0.69 (0.27, 1.78)	0.33	0.442
Stress						
Same	Ref.			Ref.		
Less				1.00 (0.35, 2.89)	0.54	0.999
More				1.29 (0.73, 2.25)	0.37	0.380
Risk-taking				**1.06 (1.01, 1.12)**	**0.03**	**0.017**
Risk-taking x Stress						
Same	Ref.			Ref.		

Less		0.95 (0.82, 1.1)	0.07	0.504
More		1.01 (0.93, 1.09)	0.04	0.834
Impatience		1.00 (0.95, 1.04)	0.02	0.859
Impatience x Stress				
Same	Ref.	Ref.		
Less		**1.17 (1.04, 1.31)**	**0.07**	**0.007**
More		1.00 (0.93, 1.08)	0.04	0.943

NS-SEC = National Statistics Socio-economic Class. Model 1: Demographics (sex, ethnicity, NS-SEC prior to the outbreak of Coronavirus, and economic activity during the pandemic). Model 2: The effect of inhibitory control (risk-taking and patience), stress, and the interaction between inhibitory control and stress, adjusting for demographics.

Table 11. Ordinal logistic regression results for the National Child Development Study with risk of alcohol-related harm due to hazardous drinking as the outcome.

	**Model 1**			**Model 2**		
**Variable**	**OR (95% CI)**	**SE**	***p***	**OR (95% CI)**	**SE**	***p***
Sex						
Male	Ref.			Ref.		
Female	**0.64 (0.52, 0.78)**	**0.07**	**< 0.001**	**0.62 (0.5, 0.76)**	**0.07**	**< 0.001**
Ethnicity						
White	Ref.			Ref.		
Non-white	**0.26 (0.12, 0.56)**	**0.10**	**< 0.001**	**0.27 (0.13, 0.58)**	**0.11**	**< 0.001**
NS-SEC analytical classes						
Higher managerial	Ref.			Ref.		
Lower managerial	0.84 (0.57, 1.24)	0.17	0.378	0.82 (0.56, 1.19)	0.16	0.295
Intermediate occupations	0.75 (0.51, 1.12)	0.15	0.160	0.75 (0.51, 1.1)	0.15	0.138
Small employers and self employed	0.71 (0.39, 1.28)	0.21	0.256	0.75 (0.42, 1.33)	0.22	0.324
Lower supervisory and technical	0.66 (0.38, 1.13)	0.18	0.133	0.71 (0.42, 1.22)	0.19	0.213
Semi-routine occupations	0.83 (0.49, 1.4)	0.22	0.483	0.83 (0.49, 1.41)	0.22	0.500
Routine occupations	**0.56 (0.33, 0.96)**	**0.15**	**0.035**	**0.55 (0.32, 0.96)**	**0.16**	**0.035**
Uncategorised	0.85 (0.52, 1.37)	0.21	0.501	0.93 (0.57, 1.52)	0.23	0.784
Economic activity						
Employed	Ref.			Ref.		
Self-employed	0.74 (0.51, 1.08)	0.14	0.120	0.66 (0.46, 0.96)	0.12	0.029
Unpaid/voluntary work	1.36 (0.67, 2.78)	0.50	0.394	1.32 (0.64, 2.73)	0.49	0.449
Apprenticeship	-	-	-	-	-	-
Unemployed	0.87 (0.37, 2.08)	0.39	0.761	0.77 (0.32, 1.82)	0.34	0.548
Permanently sick or disabled	0.59 (0.26, 1.33)	0.24	0.203	0.45 (0.2, 1.01)	0.19	0.054
Retired	1.05 (0.7, 1.59)	0.22	0.807	0.88 (0.4, 1.93)	0.35	0.750
Looking after home or family	0.76 (0.44, 1.3)	0.21	0.312	0.95 (0.62, 1.44)	0.20	0.797
In education	-	-	-	-	-	-
Uncategorised	0.92 (0.42, 2)	0.36	0.831	0.88 (0.4, 1.93)	0.35	0.750
Stress						
Same	Ref.			Ref.		
Less				0.74 (0.25, 2.16)	0.41	0.585
More				0.88 (0.49, 1.60)	0.27	0.680
Risk-taking				1.00 (0.95, 1.05)	0.03	0.945
Risk-taking x Stress						
Same	Ref.			Ref.		

Less		1.04 (0.89, 1.22)	0.08	0.631
More		1.08 (0.99, 1.18)	0.05	0.091
Impatience		1.02 (0.97, 1.06)	0.02	0.480
Impatience x Stress				
Same	Ref.	Ref.		
Less		1.04 (0.94, 1.16)	0.06	0.435
More		1.00 (0.92, 1.09)	0.04	0.972

NS-SEC = National Statistics Socio-economic Class. Model 1: Demographics (sex, ethnicity, NS-SEC prior to the outbreak of Coronavirus, and economic activity during the pandemic). Model 2: The effect of inhibitory control (risk-taking and patience), stress, and the interaction between inhibitory control and stress, adjusting for demographics.

Change in stress

Table 12. Ordinal logistic regression results for the Millennium Cohort Study with change in stress as the outcome.

**Variable**	**OR (95% CI)**	**SE**	***p***
Sex			
Male	Ref.		
Female	**1.54 (1.08, 2.20)**	**0.28**	**0.017**
Ethnicity			
White	Ref.		
Non-white	1.66 (0.75, 3.66)	0.67	0.213
NS-SEC analytical classes			
Higher managerial	Ref.		
Lower managerial	1.11 (0.26, 4.8)	0.83	0.885
Intermediate occupations	0.7 (0.17, 2.85)	0.5	0.613
Small employers and self employed	0.81 (0.13, 5.1)	0.76	0.818
Lower supervisory and technical	0.32 (0.07, 1.39)	0.24	0.128
Semi-routine occupations	0.79 (0.19, 3.29)	0.57	0.749
Routine occupations	0.55 (0.12, 2.45)	0.42	0.431
Uncategorised	0.39 (0.09, 1.67)	0.29	0.205
Economic activity			
Employed	Ref.		
Self-employed	**5.53 (1.56, 19.57)**	**3.55**	**0.008**
Unpaid/voluntary work	5.33 (0.27, 104.22)	8.05	0.269
Apprenticeship	0.54 (0.28, 1.02)	0.17	0.056
Unemployed	**1.75 (1.08, 2.83)**	**0.43**	**0.024**
Permanently sick or disabled	1.36 (0.47, 3.92)	0.73	0.567
Retired	-	-	-
Looking after home or family	0.99 (0.35, 2.8)	0.52	0.979
In education	0.39 (0.04, 3.61)	0.44	0.407
Uncategorised	0.85 (0.46, 1.59)	0.27	0.618

NS-SEC = National Statistics Socio-economic Class.

Table 13. Ordinal logistic regression results for the Next Steps cohort with change in stress as the outcome.

**Variable**	**OR (95% CI)**	**SE**	***p***
Sex			
Male	Ref.		
Female	**1.93 (1.39, 2.70)**	**0.33**	**< 0.001**
Ethnicity			
White	Ref.		
Non-white	0.93 (0.66, 1.32)	0.17	0.691
NS-SEC analytical classes			
Higher managerial	Ref.		
Lower managerial	0.80 (0.52, 1.23)	0.18	0.314
Intermediate occupations	1.26 (0.77, 2.08)	0.32	0.355
Small employers and self employed	0.48 (0.18, 1.29)	0.24	0.148
Lower supervisory and technical	0.61 (0.36, 1.02)	0.16	0.060
Semi-routine occupations	1.30 (0.79, 2.15)	0.33	0.308
Routine occupations	1.76 (0.87, 3.56)	0.63	0.114
Uncategorised	1.35 (0.68, 2.64)	0.46	0.388
Economic activity			
Employed	Ref.		
Self-employed	**2.14 (1.15, 3.98)**	**0.68**	**0.017**
Unpaid/voluntary work	2.77 (0.4, 19.05)	2.72	0.300
Apprenticeship	0.36 (0.04, 3.42)	0.41	0.375
Unemployed	1.26 (0.55, 2.91)	0.54	0.586
Permanently sick or disabled	0.57 (0.2, 1.66)	0.31	0.306
Retired	-	-	-
Looking after home or family	0.71 (0.29, 1.73)	0.32	0.452
In education	-	-	-
Uncategorised	0.87 (0.39, 1.96)	0.36	0.734

NS-SEC = National Statistics Socio-economic Class.

Table 14. Ordinal logistic regression results for the 1970 British Cohort Study with change in stress as the outcome.

Variable	OR (95% CI)	SE	*p*
Sex			
Male	Ref.		
Female	**1.62 (1.37, 1.92)**	**0.14**	**< 0.001**
Ethnicity			
White	Ref.		
Non-white	0.72 (0.4, 1.3)	0.22	0.280
NS-SEC analytical classes			
Higher managerial	Ref.		
Lower managerial	1.08 (0.86, 1.36)	0.13	0.498
Intermediate occupations	1.13 (0.88, 1.44)	0.14	0.335
Small employers and self employed	1.11 (0.74, 1.66)	0.23	0.607
Lower supervisory and technical	0.89 (0.58, 1.37)	0.19	0.602
Semi-routine occupations	1.28 (0.92, 1.78)	0.22	0.151
Routine occupations	0.92 (0.69, 1.22)	0.13	0.549
Uncategorised	1.08 (0.71, 1.63)	0.23	0.728
Economic activity			
Employed	Ref.		
Self-employed	1.21 (0.92, 1.6)	0.17	0.180
Unpaid/voluntary work	1.58 (0.36, 6.8)	1.18	0.542
Apprenticeship	-	-	-
Unemployed	1.33 (0.63, 2.8)	0.5	0.460
Permanently sick or disabled	2.07 (0.99, 4.31)	0.78	0.053
Retired	1.41 (0.86, 2.32)	0.36	0.171
Looking after home or family	0.5 (0.17, 1.44)	0.27	0.199
In education	-	-	-
Uncategorised	1.45 (0.67, 3.17)	0.58	0.348

NS-SEC = National Statistics Socio-economic Class.

Table 15. Ordinal logistic regression results for the National Child Development Study with change in stress as the outcome.

**Variable**	**OR (95% CI)**	**SE**	***p***
Sex			
Male	Ref.		
Female	**2.03 (1.66, 2.48)**	**0.21**	**< 0.001**
Ethnicity			
White	Ref.		
Non-white	0.88 (0.5, 1.56)	0.26	0.662
NS-SEC analytical classes			
Higher managerial	Ref.		
Lower managerial	1.01 (0.67, 1.51)	0.21	0.968
Intermediate occupations	1.3 (0.9, 1.87)	0.24	0.166
Small employers and self employed	0.96 (0.56, 1.64)	0.26	0.887
Lower supervisory and technical	1.58 (0.93, 2.67)	0.43	0.092
Semi-routine occupations	1.41 (0.93, 2.14)	0.30	0.110
Routine occupations	1.14 (0.67, 1.91)	0.30	0.632
Uncategorised	1.26 (0.79, 1.99)	0.30	0.336
Economic activity			
Employed	Ref.		
Self-employed	1.32 (0.94, 1.85)	0.23	0.108
Unpaid/voluntary work	1.24 (0.48, 3.21)	0.60	0.661
Apprenticeship	-	-	-
Unemployed	1.09 (0.51, 2.33)	0.42	0.818
Permanently sick or disabled	1.45 (0.79, 2.66)	0.45	0.235
Retired	1.05 (0.69, 1.6)	0.22	0.809
Looking after home or family	1.04 (0.58, 1.87)	0.31	0.884
In education	-	-	-
Uncategorised	1.60 (0.76, 3.37)	0.61	0.213

NS-SEC = National Statistics Socio-economic Class.

References

Brown, M., Goodman, A., Peters, A., Ploubidis, G. B., Sanchez, A., Silverwood, R., & Smith, K. (2020). COVID-19 Survey in Five National Longitudinal Studies: Wave 1 User Guide (Version 1). UCL Centre for Longitudinal Studies and MRC Unit for Lifelong Health and Ageing.

Falk, A., Becker, A., Dohmen, T., Enke, B., Huffman, D., & Sunde, U. (2018). Global Evidence on Economic Preferences. The Quarterly Journal of Economics, 133(4), 1645–1692. https://doi.org/10.1093/qje/qjy013

Falk, A., Becker, A., Dohmen, T., Huffman, D., & Sunde, U. (2016). An experimentally-validated survey module of economic preferences. Unpublished Manuscript, University of Bonn, Bonn, Germany., No. 9674(9674).

Kraus, L., Room, R., Livingston, M., Pennay, A., Holmes, J., & Törrönen, J. (2020). Long waves of consumption or a unique social generation? Exploring recent declines in youth drinking. Addiction Research & Theory, 28(3), 183–193.

Mackenbach, J. P., Stirbu, I., Roskam, A.-J. R., Schaap, M. M., Menvielle, G., Leinsalu, M., & Kunst, A. E. (2008). Socioeconomic Inequalities in Health in 22 European Countries. New England Journal of Medicine, 358(23), 2468–2481. https://doi.org/10.1056/NEJMsa0707519

Office for National Statistics. (2016). The National Statistics Socio-economic classification (NS-SEC). https://www.ons.gov.uk/methodology/classificationsandstandards/otherclassifications/thenationalstatisticssocioeconomicclassificationnssecrebasedonsoc2010

Office for National Statistics. (2018). Adult drinking habits in Great Britain. https://www.ons.gov.uk/peoplepopulationandcommunity/healthandsocialcare/drugusealcoholandsmoking/datasets/adultdrinkinghabits

Peltier, M. R., Verplaetse, T. L., Mineur, Y. S., Petrakis, I. L., Cosgrove, K. P., Picciotto, M. R., & McKee, S. A. (2019). Sex differences in stress-related alcohol use. In Neurobiology of Stress (Vol. 10, p. 100149). Elsevier Inc. https://doi.org/10.1016/j.ynstr.2019.100149

Probst, C., Kilian, C., Sanchez, S., Lange, S., & Rehm, J. (2020). The role of alcohol use and drinking patterns in socioeconomic inequalities in mortality: a systematic review. The Lancet Public Health, 5(6), e324–e332. https://doi.org/10.1016/S2468-2667(20)30052-9

Room, R. (2005). Stigma, social inequality and alcohol and drug use. Drug and Alcohol Review, 24(2), 143–155. https://doi.org/10.1080/09595230500102434

Room, R. (2013). Sociocultural aspects of alcohol consumption. In P. Boyle, P. Boffetta, A. B. Lowenfels, H. Burns, O. Brawley, W. Zatonski, & J. Rehm (Eds.), Alcohol: Science, Policy, and Public Health (pp. 38–46). Oxford University Press. https://doi.org/10.1093/acprof:oso/9780199655786.003.0006

Twigg, L., & Moon, G. (2013). The spatial and temporal development of binge drinking in England 2001–2009: An observational study. Social Science & Medicine, 91, 162–167. https://doi.org/10.1016/J.SOCSCIMED.2013.03.023

White, A., Castle, I. P., Chen, C. M., Shirley, M., Roach, D., & Hingson, R. (2015). Converging patterns of alcohol use and related outcomes among females and males in the United States, 2002 to 2012. Alcoholism: Clinical and Experimental Research, 39(9), 1712–1726.

Zapolski, T. C. B., Pedersen, S. L., McCarthy, D. M., & Smith, G. T. (2014). Less drinking, yet more problems: Understanding African American drinking and related problems. Psychological Bulletin, 140(1), 188–223. https://doi.org/10.1037/a0032113
